# Synergizing the Behavior Change Wheel and a Cocreative Approach to Design a Physical Activity Intervention for Adolescents and Young Adults With Intellectual Disabilities: Development Study

**DOI:** 10.2196/51693

**Published:** 2024-01-11

**Authors:** Laura Maenhout, Julie Latomme, Greet Cardon, Geert Crombez, Geert Van Hove, Sofie Compernolle

**Affiliations:** 1 Department of Movement and Sports Sciences Ghent University Ghent Belgium; 2 Department of Experimental-Clinical and Health Psychology Ghent University Ghent Belgium; 3 Department of Special Needs Education Ghent University Ghent Belgium

**Keywords:** Behavior Change Wheel, cocreation, intervention, physical activity, adolescents, young adults, intellectual disabilities

## Abstract

**Background:**

There is a need for physical activity promotion interventions in adolescents and young adults with intellectual disabilities. Current interventions have shown limited effectiveness, which may be attributed to the absence of theory and a population-specific development. Combining a planning model (including theory) and cocreation with the target audience during intervention development could potentially address this gap.

**Objective:**

This study aimed to report the systematic development of the Move it, Move ID! intervention by describing how the 8 different steps of the Behavior Change Wheel (BCW) were applied and present the results that emerged from those steps. In doing so, the (theoretical) content of the intervention is described in detail.

**Methods:**

A total of 23 adolescents and young adults (aged 14-22 years) with mild to moderate intellectual disabilities were designated as cocreators of the intervention. Across 2 groups, 6 similar cocreation sessions were organized in each. The content and sequence of the sessions were structured to align with the 8 steps of the BCW. All sessions were recorded and transcribed verbatim. Both a deductive (ie, steps of the BCW) and inductive (ie, resonating the voice of the participants) analysis approach were applied specifically focusing on identifying and describing the findings within each of the BCW steps.

**Results:**

After behavioral analysis (steps 1-4), 10 intervention goals were chosen and linked to Capability, Opportunity, and Motivation–Behavior components (theory within the BCW) that needed to be addressed. Psychological capability, social opportunity, and reflective motivation were emphasized as the first targets to focus on. A key finding was the urge for real-life social connectedness and social integration, which makes the social component as part of physical activity a central theme to focus on within intervention development. Judgments on the most suitable intervention functions (step 5) and behavior change techniques (step 7) were explained. When discussing the mode of delivery of the intervention (step 8), it was underscored that solely relying on a mobile health app would not fulfill participants’ social needs. Hence, the chosen intervention adopts a dyadic approach in which young individuals with intellectual disabilities are matched with peers without intellectual disabilities to engage in physical activities together, with a mobile app playing a supportive role in this partnership.

**Conclusions:**

The transparent description of the development process highlights why certain intervention components and behavior change techniques were chosen and how they are intertwined by means of the selected intervention design. This paper provides a detailed blueprint for practitioners wanting to integrate the BCW and its associated behavior change techniques, in combination with actively involving the target group, into their intervention development for people with intellectual disabilities.

## Introduction

### Physical Activity for People With Intellectual Disabilities

People with intellectual disabilities (IDs), defined as limitations in intellectual functioning (IQ of <70) and adaptive behavior emerging in childhood (age of <22 years) [1], are at higher risk of chronic health problems, such as type 2 diabetes, obesity, osteoarthritis, thyroid disorders, and cardiovascular diseases, than people without IDs [2-7]. In addition, compared with their peers without disabilities, individuals with IDs have less access to health care services, face increased polypharmacy, have higher poverty rates, encounter social isolation, and engage more in behaviors that put their health at risk (eg, unhealthy nutrition and physical inactivity) [4].

Promoting physical activity (PA) may be one avenue to reduce increased health problems. PA has been shown to have beneficial effects on the physical and psychosocial health of people with IDs [8,9]. Nevertheless, they are less physically active than their peers without IDs [10-16]. The World Health Organization (WHO) recommends that adolescents engage in at least 60 minutes of moderate to vigorous PA (MVPA) per day and participate in muscle- and bone-strengthening activities 3 days per week. Adults are recommended to perform at least 150 minutes of moderate-intensity PA or 75 minutes of vigorous-intensity PA throughout the week or an equivalent combination of moderate- and vigorous-intensity activity supplemented by performing activities twice a week to strengthen muscles and bones [17]. Since 2020, these global PA guidelines include groups such as people living with IDs. It is a positive trend that, for the first time, there is attention to the specific target group in the WHO PA guidelines. However, it should be noted that the evidence is primarily based on individuals without IDs, and some argue that disability-specific guidelines are necessary [2].

A 2016 systematic review including 15 studies described that only 9% of adults with IDs achieved minimum PA guidelines (with a range of 0%-46%), measured using both objective and self-reported measurement tools [18]. Different PA guidelines were used as outcome measures in the included studies, such as 150 minutes of MVPA per week (in bouts of >10 minutes), 30 minutes of MVPA for at least 5 days per week, 20 minutes of mild exercise ≥4 times per week, 12 bouts of MVPA in 4 weeks (retrospectively), and >10,000 steps per day. A systematic review the year after (2017) reported that, in 5 out of 17 studies that assessed MVPA through accelerometry in participants with IDs (aged 6-72 years), none of the participants met the PA guidelines of 150 minutes of MVPA per week for adults and 60 minutes of MVPA per day for children and adolescents. In the remaining 12 studies, the percentage of participants with IDs who met the guidelines ranged from 6% to 66% (mostly because of the use of different protocols to measure PA) [19]. Both reviews concluded that only a small number of individuals with IDs meet the PA guidelines, indicating that they are less active than the general population [18,19].

### Current PA Interventions and Their Effectiveness

Although PA research in people with IDs has been growing over the last decade, this field has been underresearched. A PA Series in *The Lancet* (2021) stated that, between 1999 and 2019, <5% of all articles in the 5 highest-impact medical journals focused on people with disabilities (not limited to IDs) and <7% of these addressed PA or health [2]. A systematic review from 2019 on the effectiveness of PA interventions among participants with IDs of all ages identified only three effective randomized controlled trials out of 9 [20]: (1) a 10-week progressive resistance training program in adolescents and young adults (aged 14-22 years) with Down syndrome in Australia [21], (2) a 12- to 16-month multicomponent diet and PA program in adults (aged 20-66 years) with mild to moderate IDs in Sweden [22], and (3) an 8-month PA and fitness program in “fast-walking” older adults with mild to moderate IDs (aged >40 years) in the Netherlands [23]. The success of these randomized controlled trials was mainly attributed to the following factors: (1) practical support from others (eg, a mentor) in guiding and helping participants with IDs through the intervention and for increasing motivation, (2) establishing a routine that involves regular PA as well as the adaptability of an intervention to the specific routines of both carers and participants, (3) the simplicity of an intervention by adapting interventions to the specific needs of the participants, and (4) familiarity with the intervention [20]. None of the 9 interventions in the systematic review by Hassan et al [20] included a technological component (ie, eHealth or mobile health [mHealth]). However, there seems to be no reason why digital interventions would not be feasible in this target group [24]. In the study by Ptomey et al [25], 95% of the participants, aged 14.9 (SD 2.2) years on average, reported that using a tablet computer was easy and enjoyable. It is then no surprise that, in recent years, there has been a growing interest in the development of digital interventions for individuals with IDs [26-30].

The number of effective PA interventions for people with IDs remains limited. A potential reason for the limited effectiveness is currently attributed to the lack of a theoretical framework for intervention development and the difficulty in concretizing behavior change techniques (BCTs) in an understandable way for this population [10,20,31,32]. A 2017 systematic review on the use of BCTs in lifestyle change interventions for people with IDs, for example, concluded that 73% of the studies aiming to improve PA in the target group did not use any theoretical framework [31]. Nevertheless, the use of a theoretical framework is an important prerequisite for intervention effectiveness [2,20,33-36]. Furthermore, when examining theory-based interventions for people with IDs, concerns have been raised regarding the suitability of the theories used (eg, social cognitive theory, theory of planned behavior, and self-determination theory) as a starting point for designing interventions for this specific target group. These theories may not sufficiently address the specific challenges faced by people with IDs. More specifically, these theories tend to be specific and detailed, yet they may not encompass the complete spectrum of potential influences on behavior within this particular target group and often concentrate on individual-level factors [20,31,32].

### Applying the Behavior Change Wheel and a Cocreational Approach to Build Theory-Based PA Interventions

The Behavior Change Wheel (BCW) is a planning model aimed at guiding a scientific and systematic intervention development process [33,37]. The BCW contains a behavioral theory at its heart, the Capability, Opportunity, and Motivation–Behavior (COM-B) model, which encompasses the full range of influences contributing to the behavior of interest [33,38]. A total of 3 behavioral components are summarized in the COM-B model, which states that, for each behavior to occur, individuals need capability (physical and psychological), opportunity (physical and social), and motivation (reflective and automatic) [33,37,39]. The COM-B model is in turn linked to the Theoretical Domains Framework (TDF) [33,40], which subdivides the COM-B model into 14 domains. The BCW further formulates 9 intervention functions linked to 93 BCTs [41] and 7 policy types with the aim of modifying each of the 3 COM-B components and, thus, changing behavior. The COM-B model describes the minimal factors that behavioral scientists agree on to achieve behavior change and has been developed with interdisciplinary research in mind [37]. It is an open model and relatively easy to communicate, especially with vulnerable groups. In recent years, this model has demonstrated applicability in the context of PA among people with IDs and their carers [32,42]. This study chose the BCW as a planning model for intervention development because of its practical use and feasibility in combination with a cocreative approach. Current lifestyle modification approaches for this target group lack a robust foundation addressing their unique needs [10,32,43]. Therefore, deeply engaging with this group and customizing approaches to promote their PA is vital. Unfortunately, individuals with IDs are seldom heard in research, and interviews with caregivers often take precedence, potentially overshadowing their authentic experiences [43]. Neglecting the perspectives of individuals with IDs can undermine intervention acceptability, comprehensibility, and feasibility [10,44-48]. To clarify, previous intervention studies have reported that some BCTs may be too complex for the target group (eg, self-monitoring through the use of pedometers) [31,49]. The cocreative approach (in combination with the BCW planning model) in this study will aid in determining which BCTs might be most appropriate for people with IDs or adapting them if necessary through collaboration.

### Aims

This paper aimed to (1) report the systematic development of the Move it, Move ID! intervention by describing how the different steps of the BCW were applied and (2) present the results that emerged from those steps. In doing so, we described the (theoretical) content of the Move it, Move ID! intervention in detail.

## Methods

### Participants and Recruitment

It was prioritized to focus on young people with IDs as cocreators rather than their parents or teachers because of the historical pattern of marginalization in previous research on intervention development [43]. Through purpose sampling, 2 class groups of adolescents or young adults with mild to moderate IDs aged between 13 and 22 years (ie, age of special needs secondary education in Flanders, Belgium) were recruited to participate in the cocreation sessions. In February 2021 and March 2021, a total of 2 physical education (PE) teachers from different special needs schools in Flanders were contacted to explain the purpose and design of the project via email and phone. They were asked whether they were interested in involving one of their classes in cocreating a PA promotion intervention. Each PE teacher subsequently suggested 1 class group to take part. All adolescents from the selected classes (classes A and B) were invited to participate during the first visit, in which written informed consent from all participants and passive consent from their parents were obtained (Table 1). Class A comprised 14 adolescents aged between 17 and 22 years with a mild to moderate level of ID (mean age 20.33, SD 1.94 years; 3/14, 21% female). Class B comprised 9 adolescents aged between 14 and 15 years with mild IDs (mean age 14.22, SD 0.44 years; 6/9, 67% girls). This aligned with cocreation guidelines, which recommend groups of 10 to 12 cocreators [45,50]. A detailed description of (the recruitment of) participants, as well as the ethical process (next subsection), can be found in the study by Maenhout et al [51].

**Table 1:** Merging the Behavior Change Wheel (BCW) with a cocreational approach.

BCW		Researchers’ tasks	Cocreation part with participants with IDs^a^
**Stage 1: understand the behavior**			
	Step 1: define the health problem in behavioral terms	Determine the health problem in behavioral terms using the literature: insufficient PA^b^ in people with IDs	No input was gathered from the participants with IDs in the first 2 steps as we relied on the literature to define the health problem and select the target behavior. Furthermore, the PI^c^ is currently affiliated with the Department of Movement and Sports Sciences (Ghent University), which is why we focused on PA.
	Step 2: select the target behavior	Select the target behavior: increasing PA levels in adolescents and young adults with IDs	No input was gathered from the participants with IDs in the first 2 steps as we relied on the literature to define the health problem and select the target behavior. Furthermore, the PIc is currently affiliated with the Department of Movement and Sports Sciences (Ghent University), which is why we focused on PA.
	Step 3: specify the target behavior and formulate intervention goals	Specify the target behavior by: Generating a nonexhaustive list of all potential barriers and facilitators that may be relevant to the target behavior Describing these barriers and facilitators as what needs to be targeted in the intervention (who needs to do it, what do they need to do differently to achieve change, where and when do they need to do it, and how often and with whom do they need to do it) Formulating 10 intervention goals based on the ranking by the cocreators	Cocreation session 1: Introduction session (ie, explanation of the project and its purpose, process of informed consent, and getting to know each other) Cocreation session 2: Comapping barriers to and facilitators of PA What PAs are they currently performing? What do they like or dislike? Cocreation session 3: Explore the most important barriers and facilitators on which the intervention should focus by voting and ranking them by importance
	Step 4: link intervention goals to COM-B^d^ components and TDF^e^ domains	Select the components of the COM-B model and the theoretical domains of the TDF for each intervention goal	No input was gathered from the participants with IDs in this step as their input (from step 3) only needed to be linked to the theoretical components of the COM-B and TDF.
**Stage 2: identify intervention options**			
	Step 5: select intervention functions	Select intervention functions using the APEASE^f^ criteria from the BCW guide [33]	No input was gathered from the participants with IDs in this step as intervention functions were first considered to be feasible by the project team. However, an open-minded perspective was adopted in which only intervention functions that were deemed not feasible by the project team were removed.
	Step 6: identify policy categories	Not applied as designers limited to a specific policy lever are directed immediately to step 7 [33]	N/A^g^
**Stage 3: identify content and implementation options**			
	Step 7: identify BCTs^h^	Choose the most appropriate BCT(s) based on the following: The BCW guide [33] Input from participants with IDs APEASE criteria (expert consultation)	Cocreation session 4: Select BCTs and identify whether selected BCTs would suit the target group or how they can be redesigned to work for them
	Step 8: identify mode of delivery	Choose mode of delivery based on the following: The literature (ie, high potential of using an mHealth^i^ intervention) Input from participants with IDs	Cocreation session 5: Explore facilitators of and barriers to mHealth How can we make an mHealth intervention as feasible and acceptable as possible for them? Cocreation session 6—this session was no longer about intervention development but about the study itself, such as the following: Explore the opinion of participants with IDs on the best recruitment strategy Find out which incentives they would prefer

^a^ID: intellectual disability.

^b^PA: physical activity.

^c^PI: principal investigator.

^d^COM-B: Capability, Opportunity, and Motivation–Behavior.

^e^TDF: Theoretical Domains Framework.

^f^APEASE: Affordability, Practicality, Effectiveness and Cost-Effectiveness, Acceptability, Side Effects or Safety, and Equity.

^g^N/A: not applicable.

^h^BCT: behavior change technique.

^i^mHealth: mobile health.

### Ethical Considerations

All participants and their parents or legal guardians received detailed and accessible information regarding the study design and purpose as well as data handling. To ensure privacy, the data were pseudonymized and only accessible to the researchers or their appointed representatives. Data confidentiality was always maintained. In consultation with the data protection officer of Ghent University (Belgium), the legal basis was changed from “active informed consent” of parents or legal guardians to “public interest,” although this did not exempt researchers from informing participants. This meant that parents or legal guardians needed to contact the researchers only if they disagreed with their child’s participation and, thus, researchers did not require active consent from parents or guardians to commence. The participants with IDs themselves were required to provide their active consent, which is why the first session involved a thorough, step-by-step review of the information and consent process with time for discussion. Young people with IDs participated voluntarily and could withdraw at any time. In appreciation of their participation, all participants received 2 cinema tickets, about which they were informed when they decided to participate. This study received approval from the Ethical Committee of the Faculty of Psychology and Educational Sciences at Ghent University, Belgium (2021/056 LM).

### Combining the BCW Development Process and a Cocreational Approach

#### Overview

**Figure 1:**
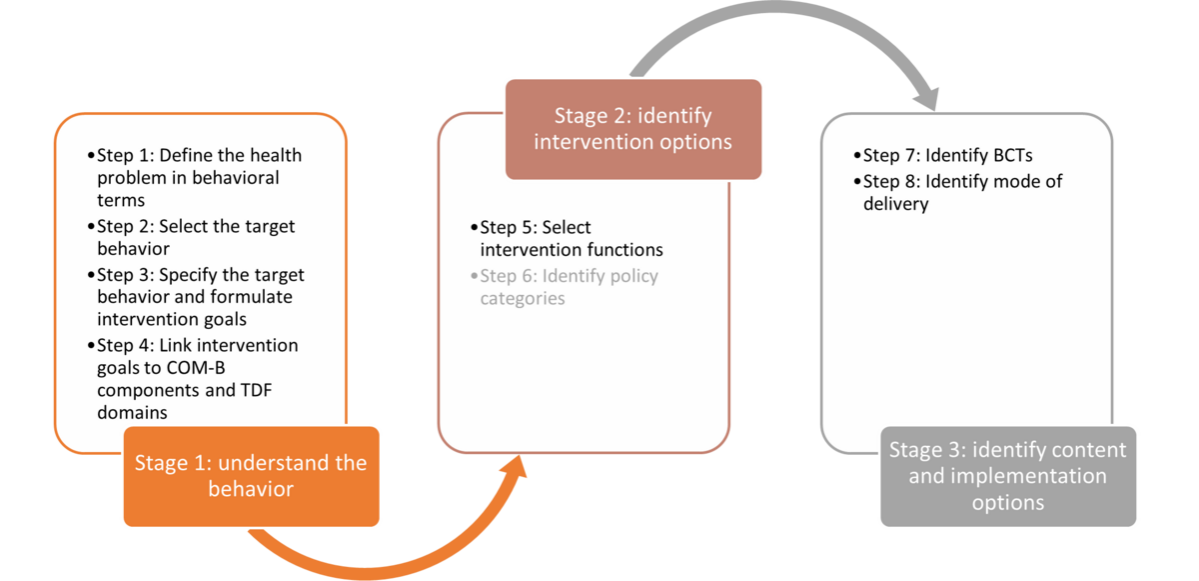
Stages of the Behavior Change Wheel: a guide to designing interventions. BCT: behavior change technique; COM-B: Capability, Opportunity, and Motivation–Behavior; TDF: Theoretical Domains Framework.

From April 2021 to June 2021, the 8 steps of the BCW were systematically followed for intervention development (Figure 1) [33]. In parallel, input was gathered from adolescents and young adults with IDs through 6 cocreation sessions (Table 1). The entire process was a mix of theoretical underpinnings (ie, COM-B), the domain expertise of the researcher, and the lived experiences of the target group (ie, cocreation sessions). The 6 sessions took place in their classrooms, each during 2 consecutive class hours. For a comprehensive explanation of the cocreation process, the methods used, and the participants’ experiences, we refer interested readers to our previously published paper [51].

#### Stage 1: Understand the Behavior

In *step 1*, the literature was reviewed by the principal investigator (PI; LM) to articulate the (health) problem in behavioral terms, this being “insufficient PA in people with IDs.” The next steps were then to select (*step 2*) and specify (*step 3*) the target behavior of the intervention, this being “increasing PA levels in adolescents and young adults with IDs,” by defining who needs to do it, what needs to be done differently to achieve change, where and when they need to do it, and how often and with whom they need to do it. This was done by generating a nonexhaustive list of possible barriers to and facilitators of PA for adolescents and young adults with IDs based on both the literature and information gathered in the second cocreation session (Table 1). Owing to the cocreative approach, insights from the literature (brought in by the PI) and input from the target group were intertwined (eg, visual cards of barriers and facilitators were developed by the PI inspired by the literature, which were brought up when the participants themselves could not come up with barriers and facilitators [anymore] [51]). In cocreation session 3, these barriers and facilitators were ranked according to importance by the target group. The most important barriers and facilitators were described as what needed to be targeted in the intervention and, consequently, formulated as the intervention goals. In *step 4*, these intervention goals were then assigned by the PI to the specific components of the COM-B model and theoretical domains of the TDF. No direct cocreation session was organized within this step as their input (from step 3) only needed to be linked to the theoretical components of the COM-B and TDF. However, this does not deviate from the essence of cocreation as the PI established these connections based on all the input provided by the participants.

#### Stage 2: Identify Intervention Options

In *step 5*, the BCW guide links COM-B components and TDF domains to 9 intervention functions [33]. Consequently, the broader research group of the PI (ie, the Physical Activity and Health research group) held expert meetings to decide which intervention functions were most suitable to work with based on the Affordability, Practicability, Effectiveness or Cost-Effectiveness, Acceptability, Side Effects or Safety, and Equity criteria [33]. These criteria are recommended by the BCW guide to make strategic judgments on the most appropriate intervention functions. No direct input from the cocreators was sought in this case, either. However, we approached this step with an open-minded perspective and only removed the intervention functions that were deemed not feasible by the project team. All other intervention functions were retained, allowing the cocreators to continue shaping the direction of development. The *sixth step* was to consider which policies would support the delivery of the intervention functions identified in step 5 [33]. However, as the researchers within this project did not have access to policy levers, step 6 was not applied. This is also described in the BCW guide by stating that “designers limited to a specific policy lever are directed to step 7 to identify BCTs” [33].

#### Stage 3: Identify Content and Implementation Options

In *step 7*, the BCW guide proposes the most appropriate BCTs for each intervention function (selected in step 5) [33]. In each of these, a distinction is made between “BCTs used most frequently and less frequently” [33]. For feasibility reasons, we focused primarily on the most frequently used BCTs during the development process. However, for the fourth cocreation session on BCTs, we also explored the less frequently used BCTs and selected relevant ones based on our expertise with the target group. The aim of this cocreation session was to find out which BCTs were understandable and feasible for adolescents and young adults with IDs and how BCTs could be adapted to meet these criteria. On the basis of the Affordability, Practicability, Effectiveness or Cost-Effectiveness, Acceptability, Side Effects or Safety, and Equity criteria and on input from the cocreators with IDs, a decision was made on which BCTs to include in the Move it, Move ID! intervention. Finally, the *eighth step* was to identify the best way to deliver the intervention (ie, mode of delivery). As research has shown that the use of technology (ie, mHealth) is feasible and has high potential in adolescents and young adults with IDs [24,25,52], the target group was asked in the fifth cocreation session about their preferences and barriers to and facilitators of mHealth use.

### Analysis

All the cocreation sessions were recorded and transcribed verbatim. A combination of a deductive (ie, 8 steps of the BCW) and inductive (ie, resonating the voice of the participants) analysis approach was applied specifically focusing on identifying and describing the findings from each of the steps of the BCW.

## Results

### Stage 1: Understand the Behavior

#### Step 1: Define the Health Problem in Behavioral Terms

Few people with IDs are sufficiently physically active [18,19].

#### Step 2: Select the Target Behavior

An increase in the total volume of PA should be targeted rather than aiming to meet the WHO guidelines regarding MVPA as even small positive changes in PA levels are associated with health benefits among people with IDs [53].

#### Step 3: Specify the Target Behavior and Formulate Intervention Goals

Multimedia Appendix 1 [32,54-63] provides an overview of 72 barriers to and 66 facilitators of PA for adolescents and young adults with IDs based on (1) a review of the literature by the PI in preparation for the cocreation sessions and (2) input from cocreators with IDs during these sessions. The appendix is divided into intrapersonal, interpersonal, and contextual factors, reflecting the multifaceted and complex nature of the influences on PA in this population. In the third cocreation session, participants ranked the barriers and facilitators according to their importance, providing guidance on which ones to address in the intervention. The 10 most important barriers (in the opposite direction, these would be facilitators) were identified: (1) the need for social connectedness, (2) the lack of practical support within the PA context, (3) the absence of a role model, (4) the need for others around them who also engage in PA, (5) the lack of confidence in their own abilities and body image, (6) the need for knowledge about the (health) benefits of PA, (7) the lack of knowledge about the different PA options available, (8) the low motivation to engage in PA, (9) the difficulty in setting goals, and (10) the need for help to incorporate PA into their existing schedules (ie, goal conflict) as they often depend on others for this. Evidently, this top list does not mean that the other barriers and facilitators were not relevant for some individuals at particular times, but in view of feasibility, it was decided to prioritize and primarily address those that were identified as the most important.

Previous studies have proposed schools as the ideal setting for PA promotion [8,32,64]. Participants with IDs in this study indicated that they are sufficiently encouraged at school to engage in PA via compulsory PE classes. However, they expressed difficulties in being physically active during leisure time. In the cocreation sessions, they expressed a preference for an intervention during their leisure time (ie, at home or in the community setting) rather than a school-based intervention:

I think it’s best to go somewhere else. Then you have something separate from school. That you are really away. When you come back to school, that you can start again with a fresh head.

Cocreator 1; cocreation session 3; group A

[...] that you just keep your activities outside school and that you don’t keep it here between these four walls.

Cocreator 2; cocreation session 3; group A

Textbox 1 summarizes the specifics of the target behavior gathered during the first 3 steps: who will perform the behavior; what needs to be done differently; and when, where, how often, and with whom it needs to be done.

Finally, the PI formulated 10 intervention goals targeting the most important barriers chosen by the cocreators (Table 2).

**Specify the target behavior (step 3 of the Behavior Change Wheel).**

**Who needs to perform the behavior?**

- Flemish adolescents and young adults aged between 14 and 22 years with mild to moderate intellectual disabilities

**What does the person need to do differently to achieve the desired change?**

- Address the 10 most important barriers or facilitators (described in Table 2)

**When do they need to do it?**

- During leisure time (weekdays+weekends)

**Where do they need to do it?**

- In the community setting or at home

**How often do they need to do it?**

- Not specified

**With whom do they need to do it?**

- Together with someone (at this stage, it was not specified yet who this someone could be, but the need for social connectedness during physical activity did emerge as the main barrier or facilitator in both groups)

**Table 2:** Linking of intervention goals to Capability, Opportunity, and Motivation–Behavior (COM-B) components and Theoretical Domains Framework (TDF) domains (step 4 of the Behavior Change Wheel).

COM-B component and relevant TDF domain				Most important barriers or facilitators		Intervention goals
**Capability**						
	**Psychological**					
		Knowledge	Insufficient knowledge about options for PA^a^, where, what suits the person best, and what are the barriers and how to counter them		Adolescents and young adults with IDs^b^ need a better understanding of where, when, and how to engage in PA; they need to be offered a range or variety of PA options they can choose from. Adolescents and young adults with IDs need a better understanding of their (own) barriers to PA and how to counter them.	
		Behavioral regulation	Difficulty in setting up PA goals (mostly because of a lack of knowledge about PA options) Difficulty with planning PA (eg, mostly because of the dependency on others and goal conflict)		Adolescents and young adults with IDs need to be facilitated/supported in formulating specific PA goals. Adolescents and young adults with IDs need to be facilitated/supported in planning PA.	
**Opportunity**						
	**Social**					
		Social influences	Lack of social connectedness; having no one to do PA with (eg, friends or loved ones) Not having a role model (ie, seeing other people engage in PA as well) No guidance during PA or no practical support		Adolescents and young adults with IDs need to have the opportunity to engage in PA together with someone (ie, social connectedness). Adolescents and young adults with IDs need a role model regarding PA. Adolescents and young adults with IDs need to have more (social and practical) support from others when engaging in PA.	
**Motivation**						
	**Reflective**					
		Intentions	No motivation to engage in PA		Adolescents and young adults with IDs need to be encouraged in feeling a sense of enjoyment when engaging in PA.	
		Beliefs about capabilities	Insecure about own capabilities and skills (eg, afraid of doing something wrong, afraid of the reaction of others, afraid of PA being too difficult, or afraid of being laughed at) Insecure or ashamed about weight or body shape		Adolescents and young adults with IDs need to build self-confidence regarding PA (ie, increase self-image and confidence in their ability to perform certain activities).	
		Beliefs about consequences	Lack of awareness about the health consequences of physical inactivity		Adolescents and young adults with IDs need a better understanding of the benefits of PA.	

^a^PA: physical activity.

^b^ID: intellectual disability.

#### Step 4: Link Intervention Goals to COM-B Components and TDF Domains

In step 4, the PI assigned the 10 intervention goals to the specific COM-B and TDF components of the BCW (Table 2). From this behavioral analysis, it can be inferred that psychological capability, social opportunity, and reflective motivation would be the first targets to focus on for increasing PA levels in adolescents and young adults with IDs.

### Stage 2: Identify Intervention Options and Steps 5 and 6 (Identify Intervention Functions and Policy Categories)

Linking the selected COM-B components and TDF domains from step 4 to the intervention functions proposed by the BCW guide, all 9 intervention functions could be applied. In total, 7 intervention functions were chosen to further focus on: education, persuasion, incentivization, training, environmental restructuring, modeling, and enablement (see the detailed argumentation in Multimedia Appendix 2). The 2 other intervention functions were not selected as they were deemed (1) less practicable to apply as a research team (ie, restriction) and (2) less acceptable or unlikely to have an impact on adolescents or young adults with IDs (ie, coercion).

### Stage 3: Identify Content and Implementation Options

#### Step 7: Identify BCTs

##### Overview

A total of 12 BCTs were selected to proceed with. We have outlined our selection and reasoning for each selected BCT within the specific intervention function in the following sections. Multimedia Appendix 3 provides a detailed explanation of all the BCTs that were considered for the 7 intervention functions that came out of step 5, along with the accompanying rationale for why they were chosen and others were not.

##### Education

Participants with IDs expressed a lack of knowledge about PA options (eg, what is out there, what suits the person best, and where can it be done). For this reason, it was considered valuable to provide information on various PA options. However, the only BCTs formulated within the taxonomy by Michie et al [41] related to providing information are pertaining to consequences (ie, social, emotional, environmental, and health). Although participants mentioned the value of information about the health benefits of PA in previous stages, we collectively decided not to place a direct emphasis on information provision within our intervention. Participants do not desire an intervention centered on “learning” or “teaching” (see also their preference for an intervention outside the school context). According to them, the focus should be on enjoyment. Nonetheless, we anticipate that the target audience may indirectly experience positive effects through the intervention. In this regard, the BCT “salience of consequences” (under the *persuasion* intervention function) seemed more applicable as it focuses on using methods to specifically emphasize the consequences of performing a behavior, making them more memorable, which goes beyond mere information provision about these consequences. “Feedback on behavior” was also selected as participants indicated that they would like to receive feedback on how well they are performing the behavior.

##### Persuasion

The BCT “credible source” was valued by participants, but opinions varied on its presentation. Some preferred health professionals using fun visual communication, whereas others liked animated movies. In this target group, experts or influencers explaining PA benefits in an engaging way were considered more appealing than scientific videos. Furthermore, the significance of “verbal persuasion about capability” was strongly emphasized. Given the low self-efficacy within this population, offering verbal persuasion to counteract self-doubt was deemed highly valuable for adolescents/young adults with IDs.

##### Incentivization

Owing to the prominent role of social factors, we observed that the BCT “social reward” would be highly motivating for this target group.

##### Environmental Restructuring

Cocreators highlighted the importance of social connectedness and support in encouraging PA. As a result, we expect the greatest impact from recognizing and meeting their social needs, which entails a “restructuring of their social environment.”

##### Modeling

Participants expressed that it would be motivating to witness others engaging in PA around them, whether in person or indirectly through influencers such as on TikTok (serving as role models). The cocreators showed enthusiasm for involving influencers they admired to encourage PA. Considering budget limitations, it would not be feasible for us to incorporate a well-known influencer into the intervention. However, this does indicate that “demonstration of the behavior” might be an interesting BCT to include.

##### Enablement

The entire development process highlighted a strong emphasis on the importance of social support and social connections, whether from friends or individuals with expertise in PA (ie, social support BCT—practical, emotional, and unspecified). Participants expressed increased confidence when they could openly discuss their goals and challenges with friends, and their motivation to engage in PA was significantly higher when they could do it with others rather than exercising alone. Peer support was generally preferred, although younger adolescents with IDs (aged 14 years) also mentioned the potential for support from family members. Furthermore, participants agreed that having a list of goals to choose from would make it easier for them rather than having to come up with their own goals (ie, goal setting BCT). Most participants recognized the importance of “action planning” as a valuable BCT. However, insights from teachers revealed that adolescents and young adults with IDs often struggle with tasks such as maintaining a personal agenda or planner, which is typically managed by parents or carers. Therefore, it would be crucial to offer guidance during action planning. Creating a detailed action plan independently, including specifics such as what, when, where, and with whom, seemed challenging and burdensome for this group. Simplicity and minimizing cognitive effort were emphasized as essential factors. Similarly, the collaborative review and adjustment of the behavioral goal with individuals with IDs based on their progress was seen as advantageous. It was considered feasible to engage in close negotiation with them to either retain the same goal, make minor adjustments, or establish a new goal if necessary (referred to as “reviewing behavior goals”). The primary focus in this case is on shared decision-making and active involvement.

##### Training

The only BCT that we considered including under the “training” intervention function is “demonstration of the behavior.” However, we view this as more related to modeling behavior rather than as actual behavior demonstration within a training context. In the course of our intervention development, it became evident that the primary focus should be on addressing social needs and creating enjoyable experiences rather than on formal training in activities. Therefore, the “training” intervention function was omitted from this phase onward.

#### Step 8: Identify Mode of Delivery

On the basis of the literature, an mHealth app appeared to be a good and feasible approach for adolescents and young adults with IDs and, therefore, was verified during the fifth cocreation session. The cocreators indicated that they preferred an mHealth app with a straightforward design that clearly indicated its purpose and functionality (eg, through an introductory video). They suggested that the app should be visually appealing, with minimal text, bright colors, and no foreign-language words. They also suggested that a game component or chat feature would be of added value. Cocreators would not use an app that they had to pay for, was childish, or looked rather old-fashioned. They mentioned preferring not to receive too many notifications (ie, no more than 1 notification per day). Finally, this is a group that often faces negative comments and experiences of failure. When talking about mHealth, this also emerged as an aspect to be considered (eg, by keeping the reactions that can be given to each other in an app controlled).

At the end of the fifth cocreation session, cocreators indicated that an app alone would not be sufficient to encourage them to engage in (more) PA. They suggested that an app could be integrated into a broader intervention but not be a stand-alone intervention. More specifically, the desire for social connection with peers and social integration in real life was found to be a more important theme in intervention development. Therefore, the decision was made to focus on a buddy system as many people with IDs reported a lack of friendships with peers outside school, resulting in decreased opportunities to engage in PA during leisure time. To facilitate this buddy partnership, we chose to work with a buddy without IDs who could offer practical support during the intervention period, which reduced the reliance on context alone (ie, parents or carers) to guide the intervention implementation.

### Move It, Move ID! Intervention

**Figure 2:**
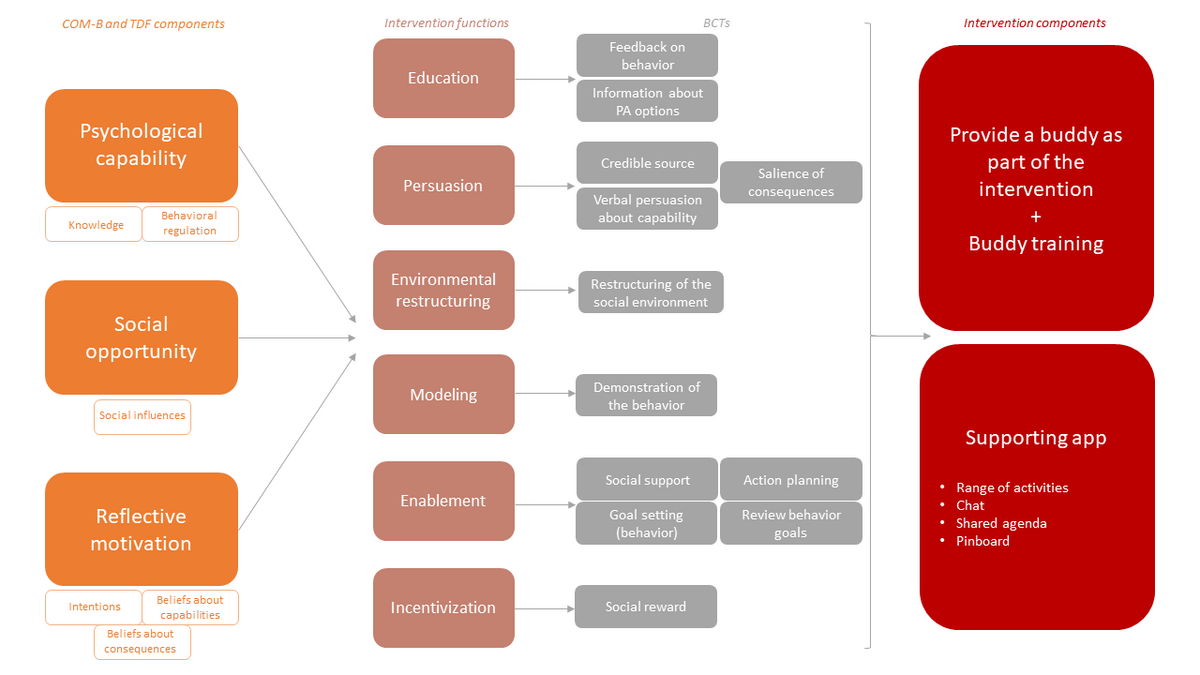
Visualization of the development process showing how Capability, Opportunity, and Motivation–Behavior (COM-B) components; intervention functions; and behavior change techniques (BCTs) are intertwined with the selected intervention design. PA: physical activity; TDF: Theoretical Domains Framework.

On the basis of the systematic steps of the BCW combined with a cocreational approach, the Move it, Move ID! intervention ultimately consists of a buddy partnership with a supporting app (ie, dyadic intervention). Figure 2 illustrates the development process, showing how COM-B components, intervention functions, and BCTs are intertwined with the selected intervention design. A more in-depth description can also be found in Multimedia Appendix 3.

During an intervention period of 3 months, adolescents and young adults with IDs will be paired with a buddy without IDs of the same age range and encouraged to try out weekly PAs in Ghent (Flanders, Belgium). Buddies without IDs will be students (aged 17-23 years) of the coauthors of this paper and will receive 3 short training sessions (ie, maximum of 1 hour per session) on their role and responsibilities as a buddy.

Although the buddy partnership forms the core of the intervention, a supporting app will also be provided in which buddies and participants with IDs will be in direct contact with each other (an explanation of the scope and screenshots of the supporting app can be found in Multimedia Appendix 4). The app is considered a private space between participants with IDs and their buddies without adding parents or carers to the app. The PI will add a range of activities (eg, walking a shelter dog, dancing, playing Kubb, and undertaking an altitude trail) to the app at the start of the intervention. Participants will have the autonomy to choose whether they want to try an activity by agreeing (swiping right) or disagreeing (swiping left) with a proposed activity. When both the participant with IDs and the buddy agree with a certain proposed activity, they will receive a pop-up to a chat function to make arrangements and schedule this activity on their shared agenda. The buddy will take the lead in this process. During an activity, the buddy can provide feedback such as how well they perform the behavior or words of encouragement. On the app pinboard, pairs can share photos of the activity they performed together, give comments, and also rate the activity afterward. This allows them to keep track of successful activities and identify less enjoyable ones.

## Discussion

### Principal Findings

This paper describes the systematic, theory-driven development of a lifestyle intervention to promote PA in adolescents and young adults with IDs using the BCW planning model combined with cocreation sessions involving the target group. The purpose of this transparent and detailed description was 2-fold. First, it aimed to develop a PA promotion intervention by identifying intervention components and BCTs that address the specific needs of this target group. Second, it aimed to encourage future researchers and intervention developers interested in PA among adolescents and young adults with IDs to apply a theoretical planning model in combination with cocreation when designing similar interventions or take the insights described into account in their own intervention development. By transparently describing the theory and BCTs that underpin the intervention, researchers are facilitated in broader evaluations to explore their driving mechanisms. In doing so, we adhered to the Medical Research Council guidelines, which emphasize the importance of theorizing how an intervention works and what works in which setting and identifying its other impacts [65]. This discussion will first delve deeper into the key findings regarding the development of the Move it, Move ID! intervention followed by a reflection on the experience of the development process by combining the BCW and cocreation.

The development process underscored the essential importance of collaborating with the target group as the intervention looks different from what the research team had envisioned in the project proposal (ie, developing an mHealth app). Active collaboration with young people with IDs highlighted the urge for real-life social connectedness and social integration, which makes the social component as part of PA a cornerstone within our intervention development [54-58]. Although the importance of social interaction has emerged in qualitative studies with the target group [66,67], this correlate has surprisingly not been included in studies examining the correlates and determinants of PA levels among young people with IDs [16,68]. However, a study from 2004 conducted within the context of the Special Olympics has already articulated that social support may be particularly crucial for individuals with IDs as they likely have a more limited friendship network compared with individuals without IDs [67]. In total, 3 intervention goals within this development process were consequently directed toward emphasizing the importance of “social opportunity” within the COM-B model. In addition, “psychological capability” and “reflective motivation” emerged as important areas for PA interventions as young people with IDs indicated a lack of knowledge about their PA options, a need for assistance in setting and planning goals, a requirement to enhance their confidence in their own capabilities, and the need to experience genuine enjoyment during PA before they would be motivated to engage in it. Throughout the remainder of the development process, the appropriate intervention functions and BCTs were then linked to these 3 COM-B components. At the end of the process, the cocreators underscored that solely relying on an mHealth app would not meet their social connectedness needs. They preferred face-to-face interaction over distant delivery modes. In addition, they expressed a preference for an intervention targeting their leisure time rather than one connected to their school context. For these reasons, a dyadic intervention was chosen in which young individuals with IDs will be paired with a peer without IDs to explore various PAs together outside the school context. A dyadic intervention refers to an approach or program that involves 2 individuals, typically with a focus on the interaction, relationship, or dynamic between them. Dyadic behavior change has been proven to be a promising approach in previous research [69-71].

As such, by incorporating an extensive and collaborative development process within a project application, one could re-evaluate the initial project proposal (ie, develop an mHealth app for young people with IDs targeted at promoting PA) with a thorough argument that adaptations are necessary from the perspective of the target group itself. In that regard, the combination of actively involving the target audience and applying a clear and scientific planning model was crucial. The most prominent planning models that are currently proposed to guide the development of effective interventions are Intervention Mapping [72] and the BCW [33]. Intervention Mapping includes 6 different steps to rigorously select determinants, performance, and change objectives using appropriate methods and strategies [72]. Although Intervention Mapping is comprehensive, its level of detail makes it more complex and, thus, less feasible, especially in combination with cocreation [73]. The BCW, in contrast, is more open, practical, and flexible as it was developed with interdisciplinary application in mind [37]. However, in applying the BCW within this project, it was noticed that its openness and flexibility could also lead to variable interpretations, with judgments from the researchers often required throughout the development process (eg, step 5). The variations in intervention development mainly depended on the resources available to the project team (eg, affordability and practicability). Moreover, even within this small research team of the Move it, Move ID! intervention, different steps within the BCW were sometimes interpreted differently. Some researchers saw the formulation of barriers to and facilitators of PA as belonging to steps 2 and 3 (as it was described in this paper), whereas others ascribed this to step 4 [74]. In our opinion, assigning these aspects to a certain step will not differ much from the behavioral diagnosis one will eventually arrive at. We consider it more important to discuss the different steps thoroughly within the research team so that the decisions made are well informed and can be argued for. By going through the different steps of the BCW, we learned that interventions can look different depending on the choices made without necessarily making one intervention better than the other. Further research should subsequently indicate which interventions prove to be effective and why (ie, identifying driving mechanisms [65]). This could potentially lead to the formulation of guidelines outlining the best possible choices that could be made during intervention development within a specific target group and setting.

Nevertheless, by applying the theoretical planning model, the PI had a clear goal in mind in setting up the structure and flow of the cocreation sessions. In doing so, the BCW was instrumental in identifying an informed behavioral diagnosis and choosing which BCTs would be most applicable to have an impact on PA behavior change within this target group and setting. Although the literature suggests that the use of theory in intervention development is key [2,20,33-36], a 2019 meta-analysis formulated that the effectiveness of interventions would be less influenced by whether they are theoretically developed than by the specific BCTs used [75]. In contrast, we believe that both (ie, theory and choice of BCTs) are intertwined. A 2017 systematic review found that lifestyle change interventions for people with IDs aimed at improving PA levels typically used 5.9 BCTs, with “provide information on consequences of behavior in general,” “plan social support/social change,” “provide instruction on how to perform the behavior,” and “goal setting (behavior)” being the most frequently used BCTs [31]. However, 73% of the studies did not use any theoretical framework for intervention development [31]. After completing the full behavioral diagnosis based on the BCW, we included 12 BCTs in our intervention. This is not to say that the inclusion of more BCTs would be better but, rather, that the transparent description of the BCW steps made more evident why these specific BCTs were chosen and how they are intertwined by means of the intervention design. This demonstrates why we believe that the use of theory and the selection of BCTs are strongly connected.

Linking cocreation to the BCW, our goal was to create an intervention that starts with the experiences of the target group. This approach was intended to enhance the effectiveness and sustainability of the intervention by making it more suitable and acceptable for the target audience [10,44-48]. Cocreation with the target audience extended well beyond the described cocreation sessions for intervention development in this project. As the project progressed toward the effect study, ongoing collaboration continued with 2 coresearchers with IDs (ie, inclusive research [48]). These coresearchers maintained regular meetings (every 2 weeks) with the PI (LM) at the Department of Movement and Sports Sciences (Ghent University), actively engaging in various facets of the project. Their responsibilities included assessing prototypes of the app; offering feedback on the training of buddies; testing measurement instruments (comprising questionnaires, interviews, and accelerometers); providing insights into the recruitment strategy; contributing to the development of promotional materials such as flyers, information letters, and informed consent forms; and participating in efforts to enhance the project’s visibility among their peers, classmates, and other stakeholders. This ongoing collaboration with the coresearchers was purposefully designed to ensure the continued accessibility of the project even beyond the initial phase of intervention blueprinting. To conclude, the described intervention development addresses an important and often overlooked population that experiences health disparities and is at higher risk of physical inactivity and related health issues. This study highlights the importance of considering the unique requirements of people with IDs to develop tailored interventions that effectively meet their needs.

### Limitations and Strengths

This study has some limitations. First, a wide age range of adolescents and young adults with IDs was included, which might make us question whether this intervention is applicable to both an individual aged 13 years and one aged 22 years. Indeed, younger adolescents with IDs (ie, aged 14 and 15 years) did indicate that they would be open to involving parents as buddies within an intervention, whereas this was not the case for young adults (ie, aged 17-22 years old). Choosing a tighter age limit (eg, ages of 13-16 years or 17-22 years) is recommended in future intervention development. Second, of the 23 cocreators, 5 (22%) had a comorbidity with autism spectrum disorder, and 1 (4%) adolescent had attention-deficit/hyperactivity disorder. This is considered a limitation as previous research has found different effects on PA among youth who have IDs and youth who have other developmental disabilities in addition to IDs [8], suggesting that further comparison of PA experiences between these groups is warranted. Within the further intervention development, little weight was given to these comorbidities. In contrast, we can also conclude that their perspective was included from the start of intervention development as they also acted as cocreators and this was not an exclusion criterion. Third, following the prioritization of young people with IDs as cocreators in the initial stages of blueprinting an intervention idea, we were unable to gather input from buddies (peers intended to be matched with the participants with IDs) and consider the broader context of individuals with IDs in the actual development phase of the intervention. This constraint was due to the project’s timeline. In light of this constraint, we recommend that future intervention developers consider including these stakeholders in subsequent phases of intervention development. Their perspectives and insights are invaluable in creating interventions that are comprehensive, inclusive, and truly reflective of the needs and dynamics of the entire participant group. The greatest strength of this study was the fact that a theoretical planning model was used in combination with cocreation to develop a PA promotion intervention for this target group. In this way, it addressed the two main reasons why current interventions often prove to be ineffective: (1) a lack of use of theory and (2) a lack of population-specific research. To the best of our knowledge, this is the first study that describes the collaborative development of a PA promotion intervention for and with adolescents and young adults with IDs. Within the Move it, Move ID! project, the decision was made to work only with participants with mild to moderate IDs; consequently, the findings cannot be extended to the target group of severe or profound IDs. Although future research should focus on the representation of all people with IDs in health research, the fact that a specific group was chosen to truly tailor an intervention to their needs can also be seen as a strength.

### Conclusions

The Move it, Move ID! intervention was developed based on the BCW in combination with cocreation. Going through this process was seen as an added value by the research team, which makes it highly recommended to allocate adequate time, budget, and experienced scientific staff for intervention development. By systematically identifying the needs of young people with IDs and linking them to theoretical concepts step by step, cocreators with IDs emphasized the importance of face-to-face interactions and social components in PA promotion interventions. They indicated that relying solely on an mHealth app would not fulfill their social needs. The intervention will consist of a dyadic approach in which young individuals with IDs are paired with a peer without IDs to engage in PAs together, with an app solely providing support within this partnership. The detailed and transparent development process described is a valuable blueprint for practitioners wanting to integrate the BCW and its associated BCTs, in combination with actively involving the target group, into their intervention development for people with IDs.

## Appendix

Multimedia Appendix 1 Barriers to and facilitators of physical activity for adolescents or young adults with intellectual disabilities.

Multimedia Appendix 2 Selection of intervention functions using the Affordability, Practicality, Effectiveness and Cost-Effectiveness, Acceptability, Side Effects or Safety, and Equity criteria.

Multimedia Appendix 3 Selection and reasoning for each selected and nonselected behavior change technique.

Multimedia Appendix 4 Scope of the Move it, Move ID! app.

## Abbreviations

BCT: behavior change technique
BCW: Behavior Change Wheel
COM-B: Capability, Opportunity, and Motivation–Behavior
ID: intellectual disability
mHealth: mobile health
MVPA: moderate to vigorous physical activity
PA: physical activity
PE: physical education
PI: principal investigator
TDF: Theoretical Domains Framework
WHO: World Health Organization

## Data Availability

The data sets generated and analyzed during this study are not publicly available to protect participants’ privacy and confidentiality because of the small number of participants but are available from the corresponding author upon reasonable request. In addition, the upcoming effect study has been preregistered at the Open Science Framework, where all other materials concerning this project can be found.
