# Supplementary material for: Synergizing the Behavior Change Wheel and a Cocreative Approach to Design a Physical Activity Intervention for Adolescents and Young Adults With Intellectual Disabilities: Development Study
Source: JMIR Form Res. 2024 Jan 11;8:e51693. doi: 10.2196/51693 (PMC10811596; doi:10.2196/51693)
Supplement: Multimedia Appendix 1 [file formative_v8i1e51693_app1.pdf]

APPENDIX 1: BARRIERS AND FACILITATORS TO PHYSICAL ACTIVITY FOR ADOLESCENTS/YOUNG ADULTS WITH INTELLECTUAL DISABILITIES

Legend:

The barriers and facilitators are ordered in a random sequence.

- Found in literature (black) [references]
- Mentioned by the participants with ID during co-creation (blue)

	Barriers	***Facilitators***
Intrapersonal factors	Insecure about own capabilities and skills (e.g., afraid of doing something wrong, afraid of PA being too difficult, afraid of being laughed at, overwhelmed by the experience of others)<br>Too shy to join a sports club<br>Lack of confidence and self-esteem in ability to perform certain activities [32]	Having and increasing confidence through PA<br>Psychological gains (e.g., increased self-image) [59, 55]
	Insecure or ashamed about weight/body shape<br>Low self-image [32]	Have the urge to look good<br>Persons seeking physical bodily changes, such as improved body composition; weight loss [32, 58]
	Difficulties with planning PA (e.g., mostly because of dependency on others and goal conflict)<br>Difficulty in setting up a goal due to a lack of knowledge about PA-options<br>Goal setting, action + coping planning and self-monitoring too difficult [59]	Action planning [59]<br>Setting goals [59]<br>Mental representations of end state that an individual wants to achieve (i.e., working towards a dream) (i.e., outcome goals)<br>Coping planning [60]
	Lack of awareness about the health benefits of physical activity<br>~ limited understanding of the importance of PA and its benefits to health [32, 58]	Feeling happy during and after exercise<br>Understanding the psychological benefits of PA [32]
	Insufficient knowledge about options for PA, where, what suits the person best, what are barriers and how to counter them, etc.<br>Lack of knowledge [56, 60]	Getting information on what the options are (i.e., knowledge)<br>Finding a kind of PA one really likes<br>Knowledge (e.g., how watching YouTube videos about exercise informed individual about exercises he should do to achieve his goal of building muscle) [54]
	No motivation/no desire to engage in PA<br>Little intention of changing existing behaviour (low PA-levels)<br>Lack of motivation [57, 60]	Motivation<br>Person's desire to be active [56]
	Preference for social media, Netflix, gaming, YouTube, etc. at home<br>Preference for sedentary activities [32, 57]<br>Preference for indoor activities [58]	Getting an incentive<br>Incentivising and rewarding PA [32, 57, 59]
	Too much stress, too much on the mind	Let go of the daily stresses of life; taking your mind off things [32]
	Low self-efficacy [54, 58]	High self-efficacy [54, 58, 61]

	Being instructed to take part in PA can make it seem like a chore [32]	Activities with an element of fun; enjoyment of PA [57, 58]<br>Gamification
	Physical limitations, e.g., reduced mobility, low motor development, low-muscle tone [32, 57, 58, 62]<br>Additional motor impairments [57]<br>Difficulties developing gross motor skills, resulting in reduced balance and coordination [32]<br>Being not completely steady on their feet [59]	Physical skills [58]<br>(Motor) skill development (which facilitated future activity) [55]<br>Physical health gains [59]:<br>* Improved flexibility [32]<br>* Increased muscle mass and bone strength [32]<br>* Developing gross motor skills [32]<br>* Improved fitness [32]
	Lack of skills [55, 56, 57, 60]	Abilities of person (in terms of physical, cognitive, social, and emotional abilities; independence level; determination and enthusiasm) [55]
	Disliking PA<br>Dislike for PA [62]	Music<br>Providing stimuli to promote engagement (e.g., music, lighting and visual aids) [32]
	Previous negative PA experiences [55, 62]	Experiences of success/previous positive experiences [55]
	Low endurance<br>Lower levels of cardiorespiratory fitness [62]	Determination to succeed [55]
	Health issues or medical concerns, such as respiratory and cardiac conditions, overweight, ear problems, etc. [32, 57]<br>Being in pain	Long-term health, general health gains [32, 62]
	Routine; regularity [57]	Routine; regular nature of PA [57]
	Behavioural problems; challenging behaviour [55, 57]	Behavioural regulation [32, 57]
	Forgetting to think about it	Step counter/sports watch (i.e., self-monitoring)<br>Self-monitoring [59-61]
	Disability (physical/cognitive) characteristics [55, 57, 58]	Tailored to individual needs [61]
	Short attention span [32]	Improvements in academic performance, productivity and concentration levels [32]
	Comprehension limitations; lack of understanding rules and concepts within play [32, 55]	Activity progression; seeing results; enabling people with ID to experience progression and competence within PA [32]
	Limited communication [32, 55, 62]	Social gains; developing socialisation skills through PA [32, 57, 59]
	Difficulties following instruction [32]	Locus of control (i.e., this refers to individuals' beliefs about to the extent to which life outcomes are determined by one's own behaviour or external factors) [54]
	Tired, no energy<br>Fatigue [32, 59]	Improved sleeping pattern [32]
	Do not like to sweat<br>Physical discomfort [57]	Understanding the physiological benefits of PA [32]

	Inability to navigate unfamiliar areas independently (i.e., parents have to go along on the route the first time)<br>Risk of getting lost [59]<br>Reduced ability to judge safety (i.e., busy roads) [55, 59]<br>No full understanding of traffic rules [59]	Autonomy over PA behaviours; autonomy to choose activities [32, 63]
	No time/already have other things to do<br>Other priorities, resistance to change in routines [57]<br>Preference for activities other than physical activities [56]	
	Fear [56, 57]; fear of failure [32]<br>Afraid of getting hurt	
	Age (because the ability gap between adolescent and typically developing peers widened) [55, 57, 62] → also not being able to play in a team anymore (physically strong enough to play with peers of the same age, but too strong to play with peers that are the same age mentally)	
	Level of ID (i.e., the more severe the level of ID, the more difficult it is to engage in PA; physical inactivity is known to increase with the severity of ID) [57]	
	Competitive elements to PA [32, 57]	
Interpersonal factors	Having no one to do PA with (e.g., friends, loved ones)<br>Lack of social connectedness (e.g., having fewer friends); lack of social network [32, 54-56, 58, 59, 62]	Being able to do physical activity together with friends/lover<br>Being part of a sports club<br>Social connectedness (e.g., building friendships and a sense of belonging; having fun with friends; sense of relatedness) [54-57]; positive social interaction with peers [58]<br>Participating with family, friends and peers can have a positive effect on PA participation [32]
	No guidance during PA/ no practical support<br>Parents are not supportive<br>Lack of sufficient support from carers/relatives; family support [54, 57, 58, 60]<br>Being dependent on others to get to places = transportation requirements [54, 59]	Being supported/coached during exercise<br>Social support [56, 61]; parental support [58]<br>Utilising additional support where appropriate (e.g., personal care assistants) [55]
	Not having a role model<br>Home life (e.g., unhealthy lifestyle behaviours of parents/carers/other family members; lack of choice in activities they participate in) = role models [54, 55]<br>Parental influence [32]	Having a role model<br>Home life (e.g., healthy lifestyle behaviours of parents/carers/other family members) = role models [54, 55]<br>Parental influence [32]
	Parents are over-protective/too worried<br>Overprotection of parents [55, 58, 62]<br>Parental concerns; parental anxiety; parents' fear about their child participating in PA (due to	Being encouraged<br>Reinforce sense of competence in PA [54]<br>Positive encouragement; reinforcement [32, 55]

	for example the negative behaviours of others towards their child (i.e., bullying; negative attitudes; prejudice)) [55, 57, 62]	
	Parental beliefs and behaviours regarding activity [55]<br>Paid carers generally have a low level of knowledge around public health recommendations on diet and PA [60]	Parents/carers understanding the benefits of activity [55]
	Friends do not engage in PA either<br>Friends/peers have power to influence and discourage PA participation [32]	Unstructured or spontaneous activity, which is generally facilitated by siblings or peers [55]
	Poor communication among carers [60]	Level of carer engagement [61]
	Drawbacks of PA with others (e.g., others have to wait for people who need even more support) [59]	Teamwork and competition in sporting activities [54]
	Lack of knowledge from teachers relating the effects of person's ID; lack of knowledge on integration → barrier to inclusion [55, 56]<br>Lack of knowledge of sports clubs and coaches required to enable person to be included, as they will require time to understand their individual needs, and to develop a relationship [32, 62]<br>Lack of staff expertise [57]	Information and education of carers [55]<br>Coach knowledge (educated in adaptive activity) [55, 56]<br>The importance of having experienced PA instructors, who help to create the environment and conditions for a fun and inclusive programme [32]<br>Caregiver's high educational level [58]
	Negative views of parents relating to their child's competence [55]	Positive parental beliefs [58]
	Being dependent on the motivation of carers [55, 59]	Staff interest (positive support) [57]
	Being dependent on the timetable of carers; time constraints parents; parents/carers having to sacrifice their own time/time demands (e.g., being a "taxi"; having to stay the duration of activity sessions; competing interests) [55, 57, 59, 62]<br>Extremely busy workloads make it difficult for teachers to find time during school, to offer additional PA opportunities, beyond the PE curriculum [32]	Time of parents/carers to provide opportunities for activity [55]<br>Ensuring family time for activity (both through home-based activity and family outings) [55]
	Prefer to meet up with friends	Wanting to be perceived as cool, fitting in with their peers [32]
	Lack of social relatedness with teachers/coaches during PA classes (may lead to negative PA experiences and feelings of frustration, negatively impacting perceived competence) [54, 62]	Positive coach-athlete relationship [58]
	Staffing levels and access to support staff (e.g., occupational therapists, physiotherapist) can restrict the types of PA available to persons with ID (i.e., due to various needs and capabilities of people with ID, support staff to assist the 'instructor' whilst delivering PA is necessary) [32, 57]	Working as a team with coaches/teachers/instructors [55]

	Societal attitudes to disability; negative societal influences (e.g., discrimination, negative attitudes and behaviours on the part of others) [55, 57]<br>Prejudice of others [62]<br>Exclusion [55]<br>Negative attitudes and stereotypes towards children/adolescents with ID [55]	Networking and communicating with physical education teachers, coaches and typically developing children [55]
	Difficult to organize family recreation activities due to the lower ability levels of their child with ID [55]	One-to-one nature [57]
	Pairing/group work can result in negative behaviour if the wrong people are placed together [32]	Positive role of siblings [58]
	Anxiety on the part of staff [57]	
Contextual/ environmental factors	Too expensive<br>High costs, limited financial resources (e.g., membership price, public transport fares, etc.) [32, 55-57, 59, 62]	Affordable activities
	Too crowdy (e.g., in a gym, in the pool, etc.)	Being able to do physical activity at home
	Unsafe environment (e.g., busy roads)	Safe environment<br>Safety (e.g., traffic) [62]
	Specific organisations for people with disabilities are too confronting<br>Lack of inclusion (e.g., segregated leisure facilities) [57]	Inclusive opportunities [55, 58]
	Transport issues (i.e., the location is too far to get there on his/her own, so parents have to bring adolescent)<br>Limited provision of transportation [32, 57, 58]	Having transport
	Lack of organised activities<br>Lack of provision of PA opportunities through after school clubs/societies; few options/not that much to choose from; lack of inclusive programmes, organised activity programmes, sports clubs and facilities [32, 55, 56, 59]	An organised activity
	Lack of variety in PA opportunities; limited options for PA [32, 57]<br>Little change of environment during PA (e.g., always at home or at the grounds of the day centre) [59]	Offering individuals a range of options [59]<br>Seek out additional opportunities for activity [55]
	Lack of material/equipment	Having the right material/equipment<br>Specialised gym equipment [55]
	Lack of space at home	Having enough space at home
	Bad weather (e.g., too cold, rainy, etc.)<br>Poor weather (e.g., winter months, cold weather or rain) [54, 55, 57-59]	Nice weather
	Geographical location [57]:<br>* Lack of disability-specific programmes in a geographical proximity [55]	Accessible facilities (in close proximity, with specialized equipment); adequate and available resources [55, 56, 58]

	* Difficult to identify inclusive clubs locally (e.g., clubs requiring lots of travel) [55, 62]<br>* Geographical barrier to "getting out" [54]	
	Lack of available information (on inclusive activities) [55]<br>A lack of information on how to conduct home-based activities [55, 62]	Information dissemination [56]
	Lack of adapted or appropriate activities [57, 58]	Adapted PA programs [58]<br>Mainstream programmes which make adaptations to people with ID [55, 56]
	Lack of safe and accessible parks [55]	Attractive environment (e.g., green)<br>Green spaces [59, 63]
	Lack of accessible activities [57, 58]:<br>* Limited access to facilities and inclusive clubs [55, 62]<br>* Demand for the PA facilities within each school can be high, meaning availability is limited [32]<br>* Limited options for inclusive clubs = lack of availability [55, 62]	Using the existing infrastructure to promote PA [32]
	Reliance on school to engage in PA [32]	Creating the opportunities to be physically active during the school day (e.g., introducing cross-curricular PA initiatives; creating the culture and policies to mandate additional PA); attending PE classes and participating PA during recess [32, 54, 58]
	Lack of clear policy guidelines in local service agencies [57]	The existence of policy guidelines concerning PA [57]
	Lack of community support (e.g., discontinued classes, lack of acceptance and awareness, high turnover among staff) [57]	Receiving support from a research team [57]
	Long transportation time to school (= less time to engage in PA) [54]	Having a pet [57]
	The MUST that comes with entering into a contract (e.g. membership in the gym, feeling obliged by being stuck to a contract)	An exergaming context [58]
	Covid-19 (i.e., co-creation sessions took place during the Covid-19 pandemic, so participants mentioned the pandemic as a barrier for them to being able to engage in PA)	TikTok (i.e., imitate and create dances)
