# Supplementary material for: Synergizing the Behavior Change Wheel and a Cocreative Approach to Design a Physical Activity Intervention for Adolescents and Young Adults With Intellectual Disabilities: Development Study
Source: JMIR Form Res. 2024 Jan 11;8:e51693. doi: 10.2196/51693 (PMC10811596; doi:10.2196/51693)
Supplement: Multimedia Appendix 2 [file formative_v8i1e51693_app2.pdf]

APPENDIX 2: SELECTION OF INTERVENTION FUNCTIONS USING THE APEASE-CRITERIA

	AFFORDABILITY	PRACTICABILITY	EFFECTIVENESS AND COST-EFFECTIVENESS	ACCEPTABILITY	SIDE-EFFECTS/SAFETY	EQUITY
**EDUCATION**<br><br>*Increasing knowledge or understanding*	Education could be provided in a budget-friendly way, for example through the use of online tools.	People involved in the intervention project should be trained to correctly inform people with ID (their abilities and needs in mind). Or material should be made available, but care should be taken then to ensure that this is the correct material and reaches the target group in the way intended.	The lack of knowledge and need for information is often mentioned in literature, so we expect that focusing on this would be a good strategy in the context of (cost-) effectiveness.	The target group expresses the need for more information on PA which allows us to assume acceptability if this would be integrated in an intervention.	We see little to no side-effects or safety-issues in applying education.	We see no disadvantages in terms of equity by increasing the knowledge of the target group, but rather advantages.
**PERSUASION**<br><br>*Using communication to induce positive or negative feelings or stimulate action*	Providing some persuasive techniques, seems feasible to us within the project budget.	This seems practically feasible to us.		Participants themselves cite that PA should be framed as 'fun' (e.g., gamification), so this seems like an acceptable intervention function to us.	We see no side-effects or safety-issues in applying persuasion.	We see no disadvantages in terms of equity by persuading people with ID to PA.
**INCENTIVISATION**<br><br>*Creating an expectation of reward*	The available budget allows little room to provide material rewards for all participants.	On a practical level (regardless of the budget), we would see no problems in providing any kind of reward.	In terms of cost-effectiveness, it does not seem interesting to us to invest in this intervention function, as the participants did indicate that it would	The co-creators preferred a material reward, such as for example being able to earn discounts, e.g. on a sports watch). Although we believe	While we are aware of the benefits of incentives (also named in literature), we can also question whether offering a reward would not	We see no disadvantages in terms of equity by applying incentivisation.

	A non-material or social reward would be an option (i.e., a badge, medal or certificate)..		be nice to receive a reward, but it would not be a must for them to be motivated.	that (given the often seen financial thresholds with this target group) even a discount would be insufficient to be able to pay for the product.<br><br>A non-material reward would be an option, but participants expressed less interest in this. It was even mentioned that a reward was not a must for them. 'Encouragement' (social reward), on the other hand, does seem important.	create the expectation of always wanting to receive a reward for participating in PA (i.e., are participants motivated for the reward or for PA per se?).	
**COERCION**<br><br>*Creating an expectation of punishment or cost*		It seems difficult to us as a research team to be able to hand out a punishment or cost when participants would be physically inactive.	Because the available literature shows that *'being instructed to take part in PA can make it seem like a chore'* is a barrier for the target group to be physically active, this intervention function seems to have little impact on the effectiveness of an intervention.	An important facilitator is to attach a 'fun' element to PA. Coercion would do the opposite and thus seems unacceptable to the target group.	Coercion could lead to a dislike for PA (e.g., participants indicating that the 'must' that comes with engaging in PA is a great barrier).	We know that individuals with ID have lower autonomy over their own life choices. Using coercion to increase PA levels would in itself increase that inequality.
**TRAINING**<br><br>*Imparting skills*	Offering (forms of) training is possible within the project budget.	If there are people available to give the training, and this can be organized within	In literature, a lack of skills (physical, social, cognitive, etc.) is often cited as a		Care must be taken to ensure that skills are taught in the right way, as well as with	Training the target group in certain skills, will positively affect the current

		the existing schedules of the participants (and their context), then this seems practically possible to us.	barrier to PA (or vice versa having them as a facilitator), so we can expect that teaching different skills to have a positive impact on effectiveness.		the necessary attention to safety.	inequity prevailing in society.
**RESTRICTION**<br><br>*Using rules to reduce the opportunity to engage in the target behaviour (or to increase the target behaviour by reducing the opportunity to engage in competing behaviours)*		Practically speaking, we as a research team seem to have little impact on adding rules in the lifeworld of people with ID.		In view of a residential setting, for example, it seems this group already lives a lot according to rules. Introducing rules around PA again, and especially if other competing, yet relevant behaviours to them, are compromised by this, it seems to us this intervention function will not be accepted by the target group.	Just as with coercion, restriction could lead to a dislike for PA.	We know that individuals with ID have lower autonomy over their own life choices. Using rules to reduce the opportunity to engage in competing behaviours would in itself increase that inequality.
**ENVIRONMENTAL RESTRUCTURING**<br><br>*Changing the physical or social context*	There are ways to apply this intervention function in a budget-friendly way.	We see possibilities within this intervention function if the environmental restructuring is not too drastic, as this does not seem practically feasible to us as a research team. We are, for example, not going to be able to physically modify the entire living environment of	During the co-creation process, several environmental barriers were listed, such as the lack of accessible facilities, the lack of material, small social network, lack of social connectedness, etc. Although these were not ranked as most important by the co-	We can ask ourselves whether the participant's context would also be open to a restructuring of the physical and/or social context. If we think of residential settings, for instance, support staff already does a lot of splendid work there, so a restructuring might be too much to ask.	Literature suggests that routine is important for this target group. Within this intervention function, we have to be careful that restructuring the physical/social context might not be too drastic for participants (and deviate too much from their current	The powerful feature we see within this intervention function is that environmental restructuring is seen as an avenue for behaviour change (rather than placing full responsibility on the individual). This fits nicely within the social model of

		participants but perhaps small adjustments are possible or we could focus on certain dynamics in the social context that could lead to positive changes.	creators to act on, we can still assume that environmental restructuring can be an effective strategy. Environmental barriers also often recur in the literature (see appendix 1).	An environmental restructuring not only has an impact on the participant with ID alone, but also on his/her wider environment (this would also have to be seen within the capabilities, opportunities and motivation of their (support) network).	routine or structure), which might lead to confusion or challenging behaviour as a possible side-effect.	disability. The medical model sees disability as an individual problem that needs to be fixed, while the social model sees it as a result of the way society is structured. In that respect, this intervention function offers a positive contribution to equity, because it assumes an environmental change rather than a purely individual one.
**MODELLING**<br><br>*Providing an example for people to aspire to or imitate*	Depending on how the modelling role would be drawn out or seen, this could be done in a budget-friendly way. Engaging a well-known person or influencer for this would be rather outside the project budget. In other words, we cannot pay people to take on a modelling role within the intervention.	Practically speaking, someone must be capable to voluntarily take on a modelling role. Alternatively, someone from the participants' own environment, if available, might be able to take this up practically.	Since influences from others (i.e., parents, wider family, friends, peers, carers, etc.) do emerge as important facilitators from literature, we might expect that this intervention function could have a positive impact on the effectiveness of an intervention.	Within the list of the most important barriers/facilitators cited, there seems to be a huge need for a role model (i.e., a person one can look up to who also values PA), so we do expect that this would be an acceptable intervention function.	On the one hand, the modelling example should model the intended behaviour (i.e., being a good role model, not someone who exhibits undesirable behaviour) and on the other hand, we should take into account that people in the lives of persons with disabilities often already show a ‘coming and going’, so we should be cautious about installing a new	We see no disadvantages in terms of equity by applying correct modelling.

					person in their lives to whom they would become attached.	
**ENABLEMENT**<br><br>*Increasing means/reducing barriers to increase capability (beyond education and training) or opportunity (beyond environmental restructuring)*	There are ways to apply this intervention function in a budget-friendly way.	This seems practically feasible. We have a research team available throughout the project period to coordinate this and provide/develop tools that can be used in a sustainable way (also after the intervention period).	The literature identifies plenty of barriers and facilitators to PA (see Appendix 1). Increasing facilitators or reducing barriers in a real world context, in every respect, could add value to effectiveness. From our point of view, a small investment in enablement can be cost-effective in the longer term.	When naming and ranking barriers/facilitators, the need for support (both practical and social) - which is seen as part of 'enablement' - was emphasised by the target group. From this, we can infer acceptability of this intervention function for the target group.	We see no side-effects or safety-issues in boosting enablement.	We see no disadvantages in terms of equity by applying enablement. On the contrary by enabling PA, it just has a positive impact on the current inequity that exists around PA and health problems compared to the general population.
**Chosen intervention functions based on the APEASE-criteria**	Based on the evaluation of the intervention functions using the APEASE-criteria, the intervention functions education, persuasion, training, environmental restructuring, modelling and enablement were chosen to further focus on.					
