# Supplementary material for: Synergizing the Behavior Change Wheel and a Cocreative Approach to Design a Physical Activity Intervention for Adolescents and Young Adults With Intellectual Disabilities: Development Study
Source: JMIR Form Res. 2024 Jan 11;8:e51693. doi: 10.2196/51693 (PMC10811596; doi:10.2196/51693)
Supplement: Multimedia Appendix 3 [file formative_v8i1e51693_app3.pdf]

APPENDIX 3: SELECTION AND REASONING FOR EACH (NON-)SELECTED BCT

Intervention function	BCTs falling under this intervention function (p. 250-254, [33])	Does the BCT meet the APEASE criteria (affordability, practicability, effectiveness/cost-effectiveness, side-effects/safety, equity) in the context of increasing PA in adolescents and young adults with ID?	Description of how these BCTs were actioned within the design and delivery of the intervention
Education	Information about social and environmental consequences<br>Information about health consequences<br>Information about emotional consequences	These BCTs appears suitable if concise information is given (participants prefer visuals or verbal cues instead of written text). Participants expressed difficulty comprehending information when processing it independently, particularly when it is presented as text. However, comprehension improves when information is explained through videos or in-person interactions, making it clearer to them. Implementing a read-aloud feature would be beneficial when information is provided in text format. Additionally, it is advisable to consolidate diverse information sources to avoid the need for searching in multiple locations.<br><br>Participants mentioned that motivational quotes could be inspiring in this context. However, they pointed out that quotes are frequently in English, and since they cannot speak English, they find them inaccessible.<br><br>Participants with ID expressed a lack of knowledge about PA options (what is out there, what suits the person best, where can it be done, etc.). For this reason, we chose to provide them information on different PA options. However, the only BCTs formulated within Michie et al.'s taxonomy [41] related to providing information pertain to information regarding social, emotional, environmental, and health consequences. Although participants mentioned the value of information about the health benefits of PA in previous stages, we collectively decided not to place a direct emphasis on information provision within our intervention. They do not desire an intervention centred on 'learning' or 'teaching' (see also their preference for an intervention outside the school context). According to them, the focus should be on enjoyment. Nonetheless, we anticipate that the target audience may indirectly experience positive effects through the intervention. In this regard, BCT 'salience of consequences' (under the persuasion intervention function) is more applicable, as it focuses on using methods to specifically emphasize the consequences of performing a behaviour, making them more memorable, which goes beyond mere information provision about these consequences.	Information about PA options was incorporated by introducing a variety of activities (e.g., walking a shelter dog, dancing, playing Kubb, hiking an altitude trail, etc.) within the app, allowing them to make selections.
	Feedback on behaviour	We consider it feasible that the participant with ID could obtain feedback on how well he/she is performing the behaviour.<br>Co-creators noted that visualizing their daily step count does not necessarily increase their motivation to take more steps. Additionally, participants mentioned that a pedometer might only provide temporary motivation.	Verbal/non-verbal feedback from their buddy during the performance of an activity can be given, such as words of encouragement on how well they perform the behaviour.

	Feedback on outcome(s) of behaviour	Co-creators indicated that monitoring and receiving feedback on their post-physical activity well-being or tracking improvements in their fitness levels would not be a priority.	Not selected
	Prompts/cues	Co-creators suggested that prompts or cues could be beneficial. For instance, receiving occasional notifications was seen as intriguing, but excessive notifications throughout the day were not preferred. It would be ideal to have the option to control notification settings. Additionally, participants emphasized that the effectiveness of prompts depends on their content. Generic prompts like "you need to move more" were found to have little to no impact. Instead, reminders related to planned activities were considered more effective, such as "don't forget you have scheduled this."	Since the target audience prefers to view a prompt as a reminder separate from prompting the actual behaviour, this BCT was not selected.
	Self-monitoring of behaviour	Some participants mentioned that using a pedometer (either through a watch or smartphone app) could facilitate increased PA. However, co-creators expressed little interest in self-monitoring within the intervention since they already have apps that automatically track steps. Furthermore, participants found the process of entering and tracking their behaviour to be burdensome and inconvenient. They also mentioned that the graphs often accompanying this feedback were difficult to understand. However, visually representing a goal, such as a chargeable battery, could be helpful to show progress toward the goal. Ideally, this representation should be automatically linked to a smartwatch or similar device to eliminate the need for manual data entry each time.	Not selected
	Self-monitoring of outcome(s) of behaviour	Monitoring behaviour, outcomes, and consequences is considered challenging for the target group. Participants themselves find it burdensome, indicating that including a BCT focused on monitoring would provide little value.	Not selected
Persuasion	Credible source	This BCT was considered suitable. However, there were differing opinions regarding the presentation of this credible source. Some individuals preferred information delivered by a health professional through visual communication in a fun manner, while others preferred an animated movie. Influencers are considered a credible source within this target group.<br>When someone with expertise explains something, such as the benefits of PA or the disadvantages of physical inactivity, in an entertaining and engaging manner, it is more appealing than a video filled with scientific knowledge that may be perceived as dull.	The buddy will be a master student from one of the authors' study programmes. The buddies will receive a short buddy training by the PI to ensure they are prepared for their role and responsibilities. As a result, the buddy can be seen as a reliable source of information.
	Information about social and environmental consequences	*See explanation of this BCT under 'education'*	*See explanation of this BCT under 'education'*
	Information about health consequences	*See explanation of this BCT under 'education'*	*See explanation of this BCT under 'education'*
	Information about emotional consequences	*See explanation of this BCT under 'education'*	*See explanation of this BCT under 'education'*
	Feedback on behaviour	*See explanation of this BCT under 'education'*	*See explanation of this BCT under 'education'*
	Feedback on outcome(s) of behaviour	*See explanation of this BCT under 'education'*	*See explanation of this BCT under 'education'*
	Focus on past success	All participants in the youngest age group expressed that this would be helpful, while also acknowledging its abstract nature.	Not selected

	Verbal persuasion about capability	Providing verbal persuasion to counteract self-doubts would be beneficial for adolescents/young adults with ID.	During the buddy training, the buddy will be trained to focus on and acknowledge the strengths and qualities of individuals with ID.
	Identity associated with changed behaviour	This BCT was presented to the participants, but proved to be far too abstract. They struggled to grasp its meaning, and as a researcher, it was challenging to practically demonstrate or provide a concrete example of this BCT.	Not selected
	Salience of consequences	The concept of capturing before and after photos was suggested, allowing individuals to visually observe the positive changes in their bodies resulting from regular PA. We decided not to include the participants' idea in the intervention as we wanted to avoid associating PA solely with weight loss (as a side effect). Our primary aim is to help the target group perceive PA as enjoyable rather than focusing on slimming.<br><br>However, this BCT was also considered in a totally different perspective than the example above. Participants do not desire an intervention centred on 'learning' or 'teaching' (see also their preference for an intervention outside the school context). According to them, the focus should be on enjoyment. Nonetheless, we anticipate that the target audience may indirectly experience positive effects through the intervention. In this regard, BCT 'salience of consequences' is more applicable, as it focuses on using methods to specifically emphasize the consequences of performing a behaviour, making them more memorable, which goes beyond mere information provision about these consequences.	On the app pinboard, pairs can share photos of the activity they did together, give comments and also rate the activity afterwards.
	Social comparison	Mixed results among participants; while some found it motivating, others believed that comparing their results with others would only increase uncertainty. On the other hand, it was mentioned that it could be enjoyable to have two participants compete against each other in small PA tasks to see who performs the best.<br><br>The teacher of one group expressed concerns about embedding social comparison in an app, stating that it would be challenging for the target group. Mapping their own PA level in the app is already cognitively demanding, and adding the aspect of comparing with others would make it even more difficult.	Not selected.
Training	Demonstration of the behaviour	Participants expressed that it would be beneficial, in terms of their self-efficacy, to observe others performing a specific activity before attempting it themselves. They find it motivating to witness others around them, whether in person or indirectly through influencers like TikTok, engaging in PA and facing challenges as well (serving as role models). They also believe it is important to present a more realistic image of bodies associated with sports and PA, as opposed to the idealized body often portrayed in society and advertising. The co-creators showed enthusiasm for involving influencers they admire to encourage PA. However, considering budget limitations, it was not feasible for us to incorporate a well-known influencer into the intervention.	The intention is for the buddy to serve as a role model and demonstrate the desired behaviour. Observing a buddy engage in a behaviour can serve as a source of inspiration, encouragement, and skill acquisition. In that regard, this BCT fits more within the 'modelling' intervention function than under the 'training' intervention function.
	Instruction on how to perform a behaviour	Co-creators expressed their insecurity about participating in PA due to the fear of being ridiculed for not performing the behaviour well or correctly. In that regard, participants could be trained to perform the behaviour well. However, in the course of our intervention development, it became evident that the primary focus should be on	Not selected.

		addressing social needs and creating enjoyable experiences rather than formal training in activities.	
	Feedback on the behaviour	*See explanation of this BCT under 'education'*	*See explanation of this BCT under 'education'*
	Feedback on outcome(s) of behaviour	*See explanation of this BCT under 'education'*	*See explanation of this BCT under 'education'*
	Self-monitoring of behaviour	*See explanation of this BCT under 'education'*	*See explanation of this BCT under 'education'*
	Behavioural practice/rehearsal	Co-creators expressed that they would feel more confident in their abilities if they had the opportunity to practice them first, preferably with someone they trust or with their friends. However, in the course of our intervention development, it became evident that the primary focus should be on addressing social needs and creating enjoyable experiences rather than formal training in activities.	Not selected
	Self-monitoring of outcome(s) of behaviour	*See explanation of this BCT under 'education'*	*See explanation of this BCT under 'education'*
	Graded tasks	The presented BCT proved to be challenging for the co-creators to envision in practical terms, resulting in limited visibility of their views on this BCT.<br>Additionally, literature suggests that graded tasks may initially result in low self-efficacy and PA behaviour but can be more beneficial in the long term after successfully mastering the behaviour [66]. Since this intervention primarily focuses on the initial promotion of PA and considering the co-creators' current low self-efficacy, we do not want to emphasize long-term mastery of specific PA behaviours using graded tasks at this stage.	Not selected
Environmental restructuring	Adding objects to the environment	If the objects fit within the budget, we find this BCT to be practically feasible. It should be noted that this BCT was not directly presented to the co-creators..	Not selected
	Prompts/cues	*See explanation of this BCT under 'education'*	*See explanation of this BCT under 'education'*
	Restructuring the physical environment	We consider this BCT less practically feasible as a research team to influence the physical environment of adolescents/young adults with ID.	Not selected
	Restructuring the social environment	Co-creators emphasized the significance of social connectedness and support in promoting PA. Therefore, we anticipate the highest effectiveness by considering and addressing their social needs.	The social environment of participants with ID is altered during the intervention period by introducing a buddy who engages in PA together with them.
Modelling	*Demonstration of the behaviour*	*See explanation of this BCT under 'training'*	*The intention is for the buddy to serve as a role model and demonstrate the desired behaviour. Observing a buddy engage in a behaviour can serve as a source of inspiration, encouragement, and skill acquisition.*
Enablement	Social support (unspecified, practical and emotional)	The need for social support and social connectedness emerged as the primary focus during the co-creation sessions, whether it be from friends or individuals knowledgeable about PA. Co-creators mentioned feeling more confident in their abilities when they could discuss their goals and challenges with friends. Additionally, they expressed a stronger motivation to engage in PA when they could participate with others rather than exercising alone. Being part of a sports club was specifically highlighted as a significant motivator, even when participants didn't initially feel like exercising. Peer support was generally preferred, although younger adolescents with ID also mentioned the possibility of support from family members.	We opted for a buddy partnership, pairing participants with ID with personal buddies without ID within the same age range. The goal is for the buddies and participants with ID to explore various PA activities together on a weekly basis in the Ghent area of Belgium, where the project will be conducted.

		Therefore, we have chosen to prioritize this as the main component of our intervention. The target group expressed a greater preference for real-life contact compared to relying on social support through technology (e.g., a chatbot or stand-alone app).<br><br>Participants mentioned the importance of getting practical help regarding PA (e.g., transportation, reminders regarding the necessary (sports) materials, etc.)	It was decided to pair participants with ID with a buddy without ID, who can offer practical support. This relieves the parents/context from taking on these responsibilities. The buddy can assist with practical matters such as finding suitable activities, locations, transportation, costs, and providing necessary materials.
	Goal setting (behaviour)	Co-creators expressed the need for support in goal setting. They preferred to be provided with options for engaging in PA, as they often found it challenging to choose from unknown options. Participants agreed that having a list of goals to choose from would make it easier for them, rather than having to come up with their own goals.<br><br>When asked about setting a group goal, such as achieving a certain number of minutes of physical activity per week as a class, the responses were mixed. It was also mentioned that for a group goal to be effective, there should be motivating factors attached to it, such as teachers having to perform a task like jumping into cold water. Concerns were raised about the potential for cheating when working towards a group goal.	To facilitate goal setting for participants, we decided to provide them with a range of options through a supporting app. The app will feature various physical activities on the home screen, such as walking with a shelter dog, biking, going to the gym, trying out boxing, and more. Participants can swipe through these activities, similar to the Tinder app, indicating whether they are interested in trying the activity or not.
	Goal setting (outcome)	Co-creators find it difficult to think about the potential positive outcomes of the desired behaviour, so we will initially focus on teaching them how to set behavioural goals within the intervention. Moreover, we want to avoid participants with ID immediately associating PA with "losing weight" (which came up several times during the co-creation sessions), as we do not want to solely focus on weight loss in relation to PA.	Not selected
	Adding objects to the environment	*See explanation of this BCT under 'environmental restructuring'*	*See explanation of this BCT under 'environmental restructuring'*
	Problem solving	This BCT was introduced to the participants, but it received little response. The teachers involved in the co-creation sessions expressed concerns that it might be too demanding for the target group to think about potential solutions in case the achievement of their goals is hindered. Formulating solutions in IF-THEN statements was deemed challenging for the participants. Additionally, literature suggests that problem solving may not be the most effective technique during the motivational phase of behaviour change, as it could lead individuals to focus on the difficulties and negatively impact their self-efficacy [67]. Co-creators nevertheless expressed it would be beneficial to learn about their own barriers to PA and how to overcome them, expecting them to do this on their own might be overly demanding.	Not selected.
	Action planning	The majority of participants identified this as an important BCT to include. However, information from the teachers revealed that planning is challenging for adolescents/young adults with ID, as they often struggle with tasks like keeping a school diary. Furthermore, it is typically parents or staff members manage the agenda of individuals with ID. Therefore, if planning activities is considered an important BCT, it seems crucial to provide guidance in this aspect. Creating a detailed action plan	After a successful activity match with their buddy in the supporting app (i.e., see 'goal setting'), participants will be directed to a chat feature where they can make practical arrangements with each other, including discussing the details of the activity such as what to do, where to go, when to meet, etc. Additionally, they will have the option to schedule the activity in the

		(including what, when, where, with whom, etc.) independently appears to be difficult and burdensome for this target group. Simplicity was emphasized as a key factor. The less cognitive effort required, the better.	shared app agenda. Buddies without ID will be instructed during the buddy training to take the lead in this.
	Self-monitoring of behaviour	*See explanation of this BCT under 'education'*	*See explanation of this BCT under 'education'*
	Restructuring the physical environment	*See explanation of this BCT under 'environmental restructuring'*	*See explanation of this BCT under 'environmental restructuring'*
	Review behaviour goal(s)	Collaboratively reviewing and adjusting the behaviour goal with the person with ID based on their progress can be beneficial. We believe it is feasible to closely work with them to either maintain the same goal, make minor modifications, or set a new goal if needed. The key emphasis here is on joint decision-making and involvement.	The buddy will regularly communicate with the adolescent/young adult with ID and their context to review the established goals, both during and after the activity. In the app, both the adolescents/young adults with ID and their buddy will have the option to rate the activity, add photos, and leave comments on the app's pin board. This allows them to keep track of successful activities and identify any less enjoyable ones. The activity rating serves as a starting point for the buddy to initiate a conversation and discuss potential adjustments to better suit the adolescent's preferences. Additionally, there will be a biweekly chat between participant with ID and the PI to discuss the intervention's progress.
	Review outcome goal(s)	Since it was decided not to set an outcome goal, it also seems irrelevant to review it.	Not selected
	Pros and cons	All participants unanimously expressed their disinterest in using this BCT as they consider it tedious to list and compare the pros and cons of participating PA.	Not selected
	Monitoring of emotional consequences	Monitoring behaviour, outcomes, and consequences is considered challenging for the target group. Participants themselves find it burdensome, indicating that including a BCT focused on monitoring would provide little value.	Not selected
Incentivisation	Social reward	Due to the prominent role of social factors, we observed that the BCT 'social reward' would be highly motivating for this target group.	The buddy provides verbal (and non-verbal) rewards if participants with ID show effort and/or progress in performing the behaviour.
