# Supplementary material for: Synergizing the Behavior Change Wheel and a Cocreative Approach to Design a Physical Activity Intervention for Adolescents and Young Adults With Intellectual Disabilities: Development Study
Source: JMIR Form Res. 2024 Jan 11;8:e51693. doi: 10.2196/51693 (PMC10811596; doi:10.2196/51693)
Supplement: Multimedia Appendix 4 [file formative_v8i1e51693_app4.pdf]

APPENDIX 4: Scope Move it, Move ID! app

The aim of the Move it, Move ID! project is to encourage young people (16-22y) with mild to moderate intellectual disabilities (ID) to be more physically active. The (web)app serves as a supporting tool within a wider intervention. The essence of the intervention is to connect young people with ID with a buddy without ID (i.e., form pairs). From that click, opportunities arise to go out and be physically active together. The various app components are explained below.

The whole app system was developed with the terms 'athlete' and 'buddy'. With 'athlete' we refer to the person with ID and with 'buddy' to the person without ID. In this way, we did not want to encrust the app with the stamp of 'intellectual disability'. The buddy without ID will know sufficiently well that he/she is the person (i.e., ‘buddy’) without ID to whom we refer. We further use the terms 'athlete' and 'buddy' to explain the different components of the app.

1) **Log-in page**: Via the back-end, the principal investigator (PI) created pairs (1 athlete and 1 buddy). To do this, the participants only needed to provide their e-mail address. Each pair received a unique email to log in via the login page and the ability to change their passwords. From that moment on, the activities of the athlete and the buddy were linked. Nobody else, outside the PI, could see what they were doing in the app.

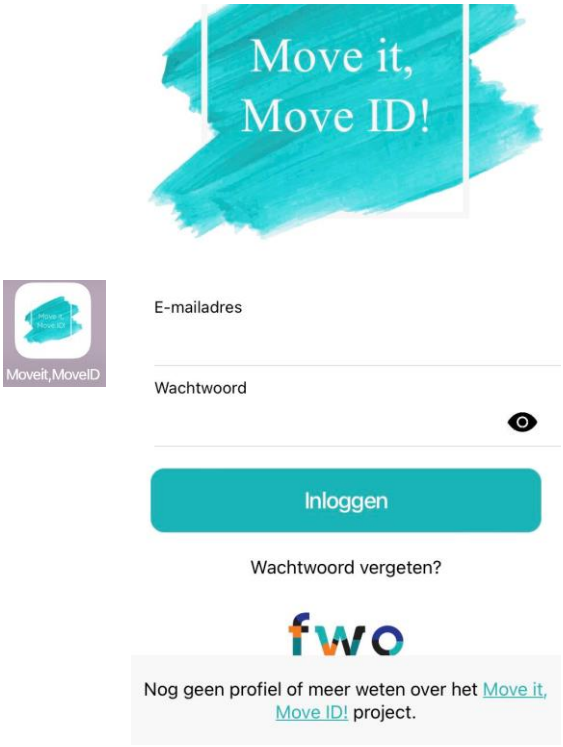

START 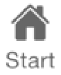

(= “home”)

2) **Activities**: On the app home screen, all kinds of possible physical activities (in the region of Ghent, Belgium) appeared that the pairs could try out together. For each activity, some extra information was provided using tags. Both athlete and buddy were able to indicate whether or

not they wanted to try out the activities via a like- or dislike-button or by swiping through the activities.

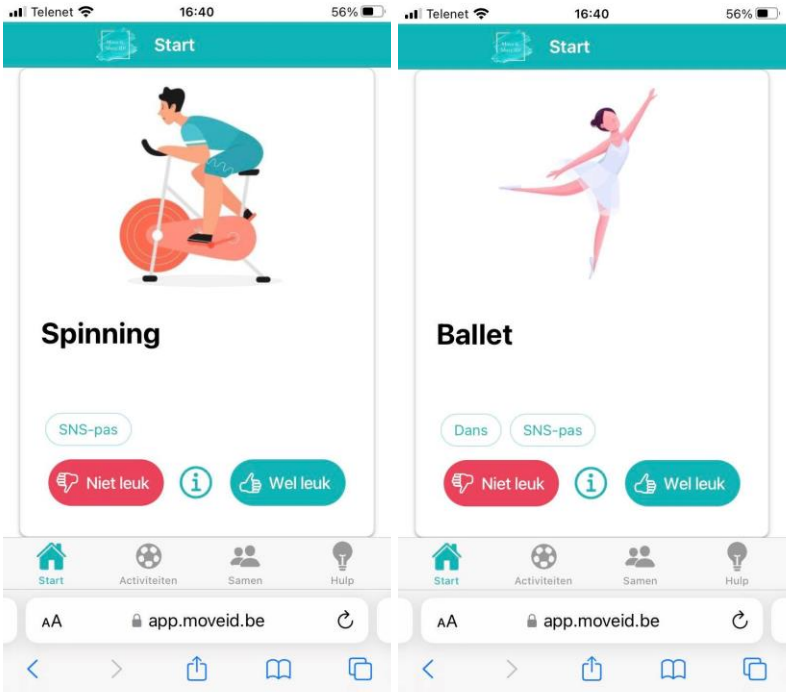

When there was a match (i.e., both the athlete and buddy wanted to try the same activity), a pop-up appeared with an immediate link to the chat function. Using the chat, participants could agree with each other when they wanted to schedule the activity.

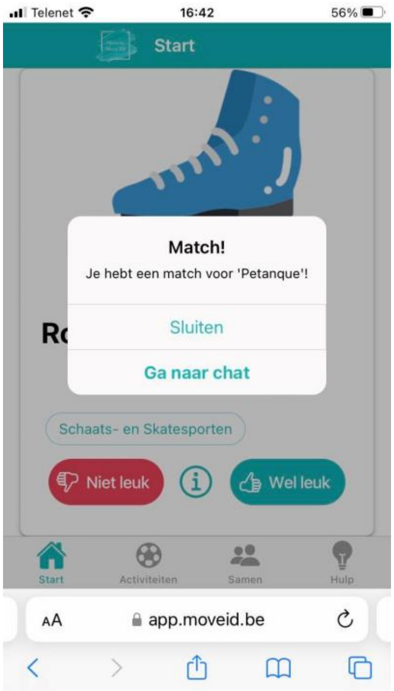

ACTIVITEITEN 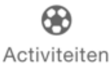 *(= "activities")*

Participants could furthermore consult which activities they 1) did not like, 2) liked but were not liked (yet) by the other person and 3) liked both. By clicking on the activity, they could find some extra information and were able to immediately schedule the activity in the agenda.

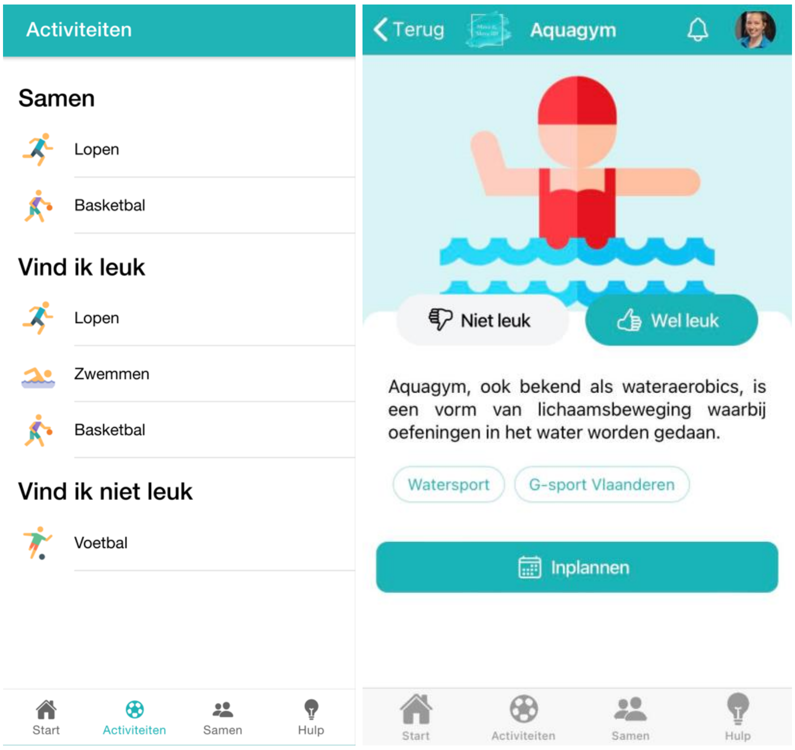

SAMEN Samen *(= "together)*

3) **Agenda**: In the agenda, participants were able to enter at what time they planned an activity. If athletes carried out an activity on their own (e.g. a walk in the woods), they could also enter this themselves using the +-button. This was then a custom-activity. This custom-activity subsequently appeared in the agenda of both the athlete and the buddy in a different colour than the activities they planned together. Conversely (i.e., the buddy scheduled in an activity he/she planned to do on his/her own and the athlete saw this via another, third colour in the agenda), we have chosen not to add this to the app. Although it might encourage the athlete when he/she sees that the buddy is planning activities separately (i.e., form of 'modelling'), we considered that buddies would probably not schedule this in the agenda of the Move it, Move ID! app, and moreover the agenda could become confusing for the athlete because of all the different colours and activities appearing. The focus was on the athlete with ID, hence only the separate and together activities of this person (via two colours) appeared in the agenda.

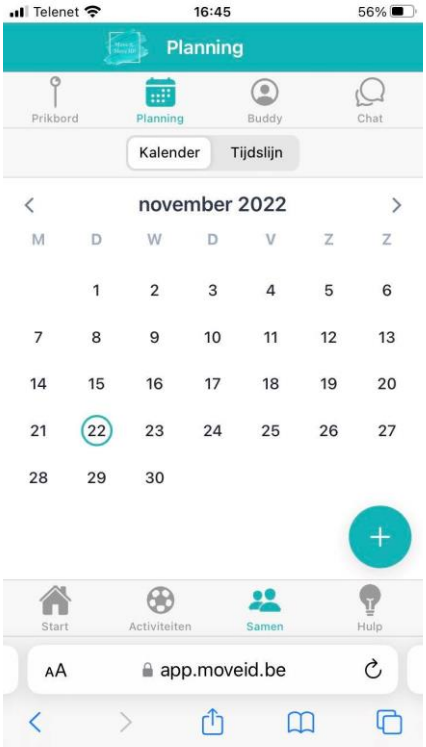

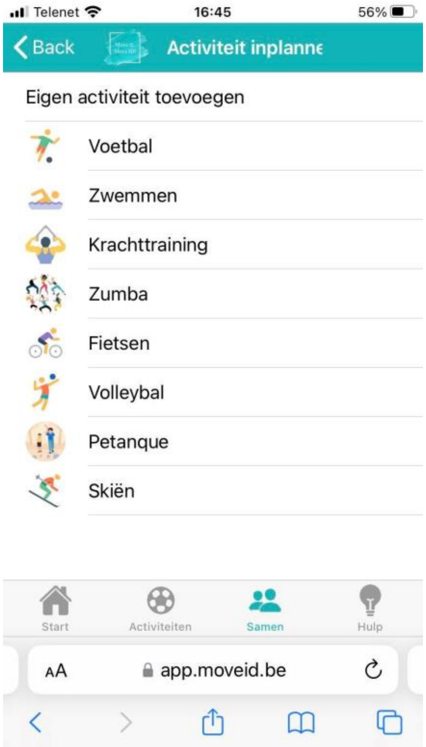

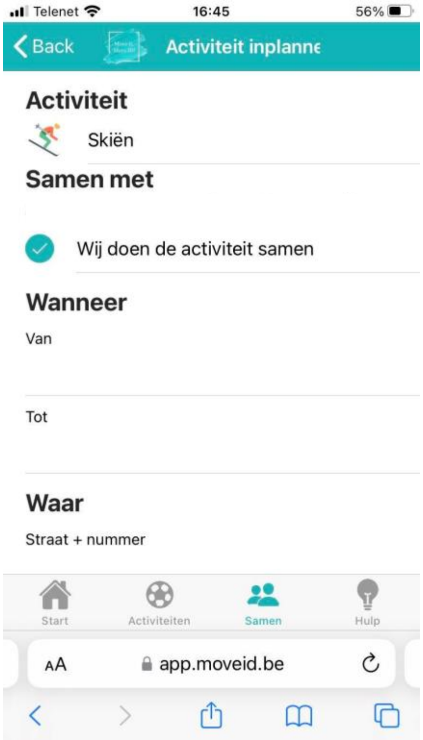

4) **Pinboard**: On the pinboard, participants could add photos and comments to their scheduled activities. In this way, the buddy could motivate the athlete when he/she was doing activities separately or pairs could save memories (e.g. photo of an activity they did together). Participants also had the possibility to evaluate the activity (using a slide of faces from not at all nice to very nice). Only the participant him-/herself (and the PI) could see that evaluation. The other person did not get to see this evaluation.

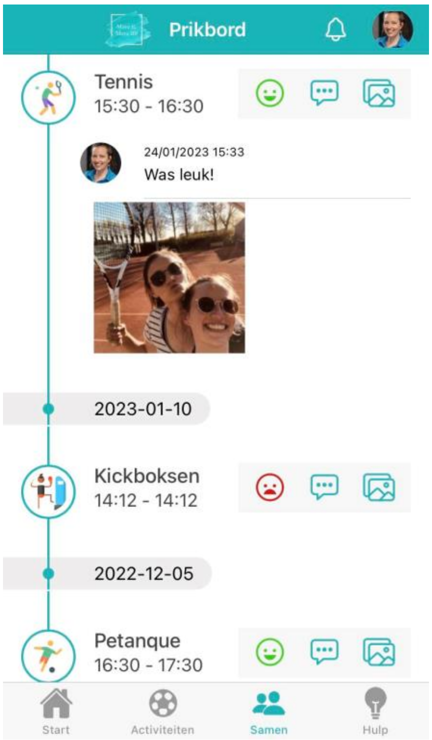

5) **Profile page**: A small profile page was integrated into the app, where both athlete and buddy could find out the name of their fellow person supplemented with a picture. The purpose was to extend this page with some more information, but due to time constraints this was not possible anymore.

6) **Chat**: Via the built-in chat, the athlete and buddy could communicate with each other. This function served mainly to make appointments for the activities and possibly to encourage each other. It was decided to build a chat function into the app for ease of use regarding making appointments, but also because this way no telephone numbers need to be exchanged. From our own experience with the target group, we learned that some young people with ID can be rather dependent, and would dare to overload people with messages (out of a need for socialising). Via the chat function, contact between the athlete and buddy could stay limited to the app. There was sufficient control over the chat function, as the PI could follow it through the back-end. If there were any problems, it was possible to intervene in time.

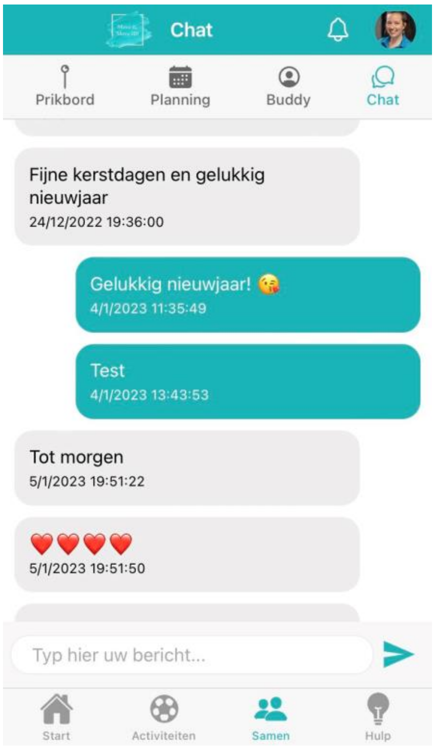

HULP 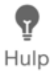

(= "help")

7) **Support page**: It was the purpose to add an introduction video to this page whereby it would be explained via screencast how to get started with the app. Such an introductory video could help if participants were not yet completely familiar with how the app worked. Due to time constraints, we could only include the contact information of the PI to make sure participants had the possibility to contact the PI easily in case of questions or problems.
